# Exercise Training and Weight Gain in Obese Pregnant Women: A Randomized Controlled Trial (ETIP Trial)

**DOI:** 10.1371/journal.pmed.1002079

**Published:** 2016-07-26

**Authors:** Kirsti Krohn Garnæs, Siv Mørkved, Øyvind Salvesen, Trine Moholdt

**Affiliations:** 1 Department of Circulation and Medical Imaging, Medical Faculty, Norwegian University of Science and Technology (NTNU), Trondheim, Norway; 2 Department of Public Health and General Practice, Medical Faculty, Norwegian University of Science and Technology (NTNU), Trondheim, Norway; 3 Clinical Service, St. Olavs Hospital, Trondheim University Hospital, Trondheim, Norway; 4 Department of Women’s Health, St. Olavs Hospital, Trondheim University Hospital, Trondheim, Norway; University of Cambridge, UNITED KINGDOM

## Abstract

**Background:**

The effectiveness of exercise training for preventing excessive gestational weight gain (GWG) and gestational diabetes mellitus (GDM) is still uncertain. As maternal obesity is associated with both GWG and GDM, there is a special need to assess whether prenatal exercise training programs provided to obese women reduce the risk of adverse pregnancy outcomes. Our primary aim was to assess whether regular supervised exercise training in pregnancy could reduce GWG in women with prepregnancy overweight/obesity. Secondary aims were to examine the effects of exercise in pregnancy on 30 outcomes including GDM incidence, blood pressure, blood measurements, skinfold thickness, and body composition.

**Methods and Findings:**

This was a single-center study where we randomized (1:1) 91 pregnant women with a prepregnancy body mass index (BMI) ≥ 28 kg/m^2^ to exercise training (*n =* 46) or control (standard maternity care) (*n =* 45). Assessments were done at baseline (pregnancy week 12–18) and in late pregnancy (week 34–37), as well as at delivery. The exercise group was offered thrice weekly supervised sessions of 35 min of moderate intensity endurance exercise and 25 min of strength training. Seventeen women were lost to follow-up (eight in the exercise group and nine in the control group). Our primary endpoint was GWG from baseline testing to delivery. The principal analyses were done as intention-to-treat analyses, with supplementary per protocol analyses where we assessed outcomes in the women who adhered to the exercise program (*n =* 19) compared to the control group. Mean GWG from baseline to delivery was 10.5 kg in the exercise group and 9.2 kg in the control group, with a mean difference of 0.92 kg (95% CI −1.35, 3.18; *p =* 0.43). Among the 30 secondary outcomes in late pregnancy, an apparent reduction was recorded in the incidence of GDM (2009 WHO definition) in the exercise group (2 cases; 6.1%) compared to the control group (9 cases; 27.3%), with an odds ratio of 0.1 (95% CI 0.02, 0.95; *p =* 0.04). Systolic blood pressure was significantly lower in the exercise group (mean 120.4 mm Hg) compared to the control group (mean 128.1 mm Hg), with a mean difference of −7.73 mm Hg (95% CI −13.23, −2.22; *p =* 0.006). No significant between-group differences were seen in diastolic blood pressure, blood measurements, skinfold thickness, or body composition in late pregnancy. In per protocol analyses, late pregnancy systolic blood pressure was 115.7 (95% CI 110.0, 121.5) mm Hg in the exercise group (significant between-group difference, *p =* 0.001), and diastolic blood pressure was 75.1 (95% CI 71.6, 78.7) mm Hg (significant between-group difference, *p =* 0.02). We had planned to recruit 150 women into the trial; hence, under-recruitment represents a major limitation of our results. Another limitation to our study was the low adherence to the exercise program, with only 50% of the women included in the intention-to-treat analysis adhering as described in the study protocol.

**Conclusions:**

In this trial we did not observe a reduction in GWG among overweight/obese women who received a supervised exercise training program during their pregnancy. The incidence of GDM in late pregnancy seemed to be lower in the women randomized to exercise training than in the women receiving standard maternity care only. Systolic blood pressure in late pregnancy was also apparently lower in the exercise group than in the control group. These results indicate that supervised exercise training might be beneficial as a part of standard pregnancy care for overweight/obese women.

**Trial Registration:**

ClinicalTrials.gov NCT01243554

## Abstract 2

Moholdt and colleagues show that exercise training in pregnancy reduces incidence of gestational diabetes and blood pressure, but not weight gain.

## Author Summary

**Why Was This Study Done?:**

- Maternal obesity is associated with increased risk of several adverse pregnancy outcomes.
- The aim of our study was to investigate whether exercise training during pregnancy can reduce gestational weight gain and prevent negative health outcomes, such as gestational diabetes mellitus and high blood pressure, among overweight/obese pregnant women.

**What Did the Researchers Do and Find?:**

- Ninety-one overweight/obese pregnant women were randomly allocated to an exercise group or a control group in early pregnancy (starting in pregnancy week 12–18).
- Women in the exercise group were asked to attend supervised sessions of combined endurance and resistance training three times weekly.
- Women in both groups gained on average about 10 kg. In late pregnancy, there seemed to be fewer women in the exercise group with gestational diabetes mellitus, and the exercising women had lower systolic blood pressure compared to those in the control group.

**What Do These Findings Mean?:**

- Providing an exercise program to overweight/obese pregnant women did not reduce gestational weight gain compared to standard pregnancy care.
- Exercise training might reduce the incidence of gestational diabetes mellitus and lower systolic blood pressure in late pregnancy in this population.

## Introduction

Maternal obesity is a risk factor for adverse pregnancy outcomes, such as gestational diabetes mellitus (GDM) [1], gestational hypertension, preeclampsia, need for cesarean delivery, and large for gestational age [2–4]. Because the prevalence of overweight and obesity among reproductive-age women is increasing, effective preventive strategies are urgently needed.

Excessive gestational weight gain (GWG) is also associated with negative obstetric outcomes [1,5,6]. The 2009 Institute of Medicine (IOM) guidelines on GWG suggest that underweight women (body mass index [BMI] ≤ 18.5 kg/m^2^) should gain 12.5–18.0 kg during pregnancy; normal weight women (BMI 18.5–24.9 kg/m^2^), 11.5–16.0 kg; overweight women (BMI 25.0–29.9 kg/m^2^), 7.0–11.5 kg; and obese women (BMI ≥ 30.0 kg/m^2^), 5.0–9.0 kg [7]. Overweight and obese women are about two times more likely than normal weight women to exceed these recommendations [8]; thus, there is a special need to find feasible and effective interventions to reduce GWG in women with a high BMI.

Previous research on clinical effects of lifestyle interventions during pregnancy in overweight/obese women has shown conflicting results [9–14]. Most studies have assessed the combined effect of physical activity and dietary guidance. To our knowledge, there are only three previous randomized controlled trials (RCTs) [14–16] assessing the isolated effects of exercise training in pregnancy on GWG and clinical outcomes in overweight and obese women. These studies found no significant difference in GWG between exercise and control groups. However, one study was limited by a small study sample (*n =* 12) [14], and one study reported results from only a subgroup [15].

Few studies exist on GDM prevention via exercise training in obese women [17–19], and to our knowledge no previous RCT has shown that GDM can be prevented by exercise training as the sole intervention [14,18,20,21]. However, according to a recent meta-analysis [22], structured physical exercise programs during pregnancy decrease the risk of GDM. Hence, there is still a need to establish the potential effects of exercise training on GDM prevention, and especially so in overweight/obese women.

To address the shortcomings in the research on effective prevention of GWG and of GDM, our aim was to assess whether regular supervised exercise training could reduce GWG and improve clinical outcomes, compared to standard maternity care, in women with a prepregnancy BMI of 28 kg/m^2^ or more.

## Methods

### Design and Participants

The study was approved by the Regional Committee for Medical and Health Research Ethics (REK midt 2010/1522) and registered in ClinicalTrials.gov (NCT01243554). The Exercise Training in Pregnancy (ETIP) trial was a single-center, parallel-group RCT of regular exercise training during pregnancy compared to standard maternity care in women with prepregnancy BMI ≥ 28 kg/m^2^. The study protocol has been published previously [23]. The trial was performed at the Norwegian University of Science and Technology (NTNU) and St. Olavs Hospital, Trondheim University Hospital, in Trondheim, Norway.

We made the following changes to the protocol after trial commencement: body composition was measured using air displacement plethysmography starting 28 June 2011, to improve assessments of body composition. The time limit for completed baseline testing and inclusion into the study was changed from gestational week 16 to gestational week 18 on 15 November 2012, and we changed the inclusion criteria for BMI from ≥30 to ≥28 kg/m^2^ on 22 March 2013. We changed the time limit for inclusion and the BMI criteria to increase recruitment into the trial. All changes were reported and approved by the Regional Committee for Medical and Health Research Ethics. The procedures followed in the ETIP study were in accordance with ethical standards of research and the Helsinki Declaration.

At recruitment, women received written information, and they signed informed consent on behalf of themselves and their offspring before participation and randomization. Inclusion criteria were prepregnancy BMI ≥ 28 kg/m^2^, age ≥ 18 y, gestational week < 18, and carrying one singleton live fetus at 11–14 wk ultrasound scan. The participants had to be able to come to St. Olavs Hospital for assessments and exercise classes. Exclusion criteria were high risk for preterm labor, diseases that could interfere with participation, and habitual exercise training (twice or more weekly) in the period before inclusion. Women were recruited through invitations sent along with notices for routine ultrasound scan at the hospital, and additionally through Google advertisements. The women received infant food worth US$65. The participants in this study gave written informed consent to publication of their case details.

### Intervention and Outcomes

The exercise group was offered, in addition to standard maternity care, exercise sessions at the hospital three times weekly, from baseline (gestational week 12–18) until delivery. The exercise sessions were supervised by a physical therapist and were in accordance with the recommendations from the American College of Obstetricians and Gynecologists [24]. Each session lasted 60 min and consisted of treadmill walking/jogging for 35 min (endurance training) and resistance training for large muscle groups and the pelvic floor muscles for 25 min. The intensity of the endurance training was set to ~80% of maximal capacity (corresponding to Borg scale 12–15) [25]. The resistance training consisted of squats, push-ups, diagonal lifts on all fours, and oblique abdominal crunches, with three sets of ten repetitions of each exercise separated by a 1-min rest between sets. Participants also did three sets of the “plank exercise” for 30 s. We adjusted the program according to each woman’s strength level. The pelvic floor exercises consisted of three sets of ten repetitions of pulling the pelvic floor up and holding the contraction for 6–8 s.

In addition, the women were asked to follow a 50-min home exercise program at least once weekly (35 min of endurance training and 15 min of strength exercises) and to do daily pelvic floor muscle exercises. We registered adherence to the supervised exercise program, and the participants reported their home exercise in a training diary. The participants received a weight gain curve showing recommended weight gain throughout pregnancy in accordance to 2009 IOM guidelines [7], and were encouraged to compare their own weight gain with this curve. The women were invited to attend one motivational interview session [23], either individually or in a group, during the intervention period.

The control group received ordinary maternity care by their midwife, general practitioner, and/or obstetrician. The Norwegian national directions for standard maternity care among healthy pregnant women at the time the study was conducted included offering of an ultrasound examination by gestational week 18 and providing information about healthy eating and healthy lifestyle [26]. The women in the control group were asked to continue their normal daily activities and were not discouraged from exercising on their own.

All participants underwent the same test protocol at baseline (gestational week 12–18) and at late pregnancy (gestational week 34–37). In addition, the hospital personnel measured the women’s body weight immediately before delivery.

Our primary outcome measure was GWG calculated as the difference between weight at baseline and weight at delivery. Maternal body weight at baseline, in late pregnancy, and before delivery was measured with a calibrated electronic scale (Seca 770, Medema, Norway) to the nearest 0.1 kg, with the participant wearing indoor clothing, without shoes. If the hospital staff did not have time to measure the women’s weight right before delivery, we used women’s self-reported weight at the time of delivery to calculate the outcome measure.

Secondary outcome measures were BMI, body composition, physical activity level, skinfold thickness, blood pressure, various blood tests, incidence of GDM, and incidence of maternal hypertension in late pregnancy. Height was measured at baseline with a wall-mounted Seca 222 stadiometer. BMI was calculated as weight in kilograms divided by the square of height in meters. Systolic and diastolic blood pressure were measured on the right arm after 15 min of supine resting using a CASMED 740 MAXNIBP (CAS Medical Systems). We used the average of three measurements taken at 2-min intervals. Skinfold thickness was measured on the right side of the body at the sites subscapular, biceps, and triceps, using a Harpenden Skinfold Caliper (Holtain). We used the average of three measurements for each site. Body composition was measured using air displacement plethysmography (BOD POD, COSMED). The participant entered the BOD POD wearing only underwear and a swim cap. Physical activity level was measured by a questionnaire where the participants reported their frequency, duration, and intensity of weekly physical activity.

After a 10-h fast, we drew venous blood for fasting plasma glucose and other blood measurements. The participants then drank 75 g of glucose dissolved in 2.5 dl of water, and blood was drawn again after 2 h (120-min plasma glucose). According to the study protocol [23], GDM was to be diagnosed by the 2009 WHO definition: fasting plasma glucose ≥ 7.0 mmol/l and/or 120-min plasma glucose ≥ 7.8 mmol/l [27]. However, in 2013 WHO, in collaboration with the International Association of Diabetes and Pregnancy Study Groups (IADPSG), endorsed adjusted diagnostic criteria for classification of GDM: fasting plasma glucose ≥ 5.1 mmol/l and/or 120-min plasma glucose ≥ 8.5 mmol/l [28]. GDM is therefore reported here by both definitions. Plasma glucose, high-sensitivity C-reactive protein (CRP), total cholesterol, high-density lipoprotein (HDL) cholesterol, low-density lipoprotein (LDL) cholesterol, triglycerides, HbA1c, ferritin, and hemoglobin were measured using a Roche Modular P. We assessed insulin with ELISA (IBL International) using a DS2 ELISA processing system (Dynex Technologies). All assays were performed according to the manufacturer’s instructions. The inter- and intra-assay coefficients of variation were 2.1% and 1.5% for glucose, 3.8% and <1% for high-sensitivity CRP, 2.5% and 0.9% for total cholesterol, 2.8% and 0.8% for HDL cholesterol, 2.4% and 0.8% for LDL cholesterol, 2.9% and 0.9% for triglycerides, and 5.3% and 9.5% for insulin. Homeostatic assessment of insulin resistance (HOMA2-IR) was calculated as [glucose × insulin]/22.5 [29].

### Statistical Methods

Sample size was calculated based on prior studies [30,31] using a 6-kg clinically relevant difference in mean weight gain between the exercise and the control group, from baseline to delivery. According to this, a two-sided independent sample *t*-test with a 5% level of significance, a standard deviation of 10, and a power of 0.90 gave a target study population of 59 in each group. Dropout was estimated at 15%; therefore, we aimed to include 150 women.

After baseline assessments, the participants were randomly allocated 1:1 to the intervention or the control group. Allocation was done using a computer random number generator developed and administrated at the Unit for Applied Clinical Research, NTNU. The randomization had varying block sizes, with the first, the smallest, and the largest block defined by the computer technician at the Unit for Applied Clinical Research. The investigators enrolling the patients (K. K. G. and T. M.) got the allocation results on screen and by e-mail after registration of each new participant into the study and did not have the full randomization list available.

Weight measurement at delivery and blood analyses were done by personnel blinded for group allocation. All other assessments and intervention administration were done non-blinded. The statistician conducting the statistical analyses was blinded for group allocation.

The trial and the principal analyses were based on intention to treat. All available data were used at all time points. We also performed, as described in the original protocol, per protocol analyses including only the women in the exercise group who adhered to the exercise protocol [23]. Baseline data were tested for normality and analyzed by an independent sample *t*-test and by Fisher’s exact test.

The outcome measurements were analyzed in accordance to the treatment arm to which patients were randomized, regardless of nonadherence. The effect of treatment on the primary and secondary outcomes was assessed with mixed linear models for continuous outcomes and mixed logistic models for dichotomous outcomes. For the primary outcome, the effect of time and treatment was taken as a fixed effect having the levels baseline, training late pregnancy, control late pregnancy, training delivery, and control delivery. For the secondary outcomes, the effect of time and treatment was taken as a fixed effect having the levels baseline, training late pregnancy, and control late pregnancy. Due to randomization, no systematic differences between groups at baseline were assumed. To account for repeated measurements, participant ID was included as a random effect. The analyses were performed using R version 2.13.1, Stata version 13.1, and IMB SPSS Statistics 22. All results are given as mean values with 95% confidence intervals, and *p*-values less than 0.05 were considered significant. We did supplementary analyses of GWG where we adjusted for gestational age at delivery.

Per protocol analyses [23] including only the women in the exercise group who adhered to the exercise protocol were performed on both primary and secondary outcomes. Adherence to the exercise protocol was defined as (1) attending ≥ 42 organized exercise sessions, (2) attending ≥ 28 exercise sessions + performing ≥ 28 home exercise sessions, or (3) performing ≥ 60 home exercise sessions. The exercise had to be ≥50 min of either aerobic or strength training to count as a home session.

## Results

**Fig 1:**
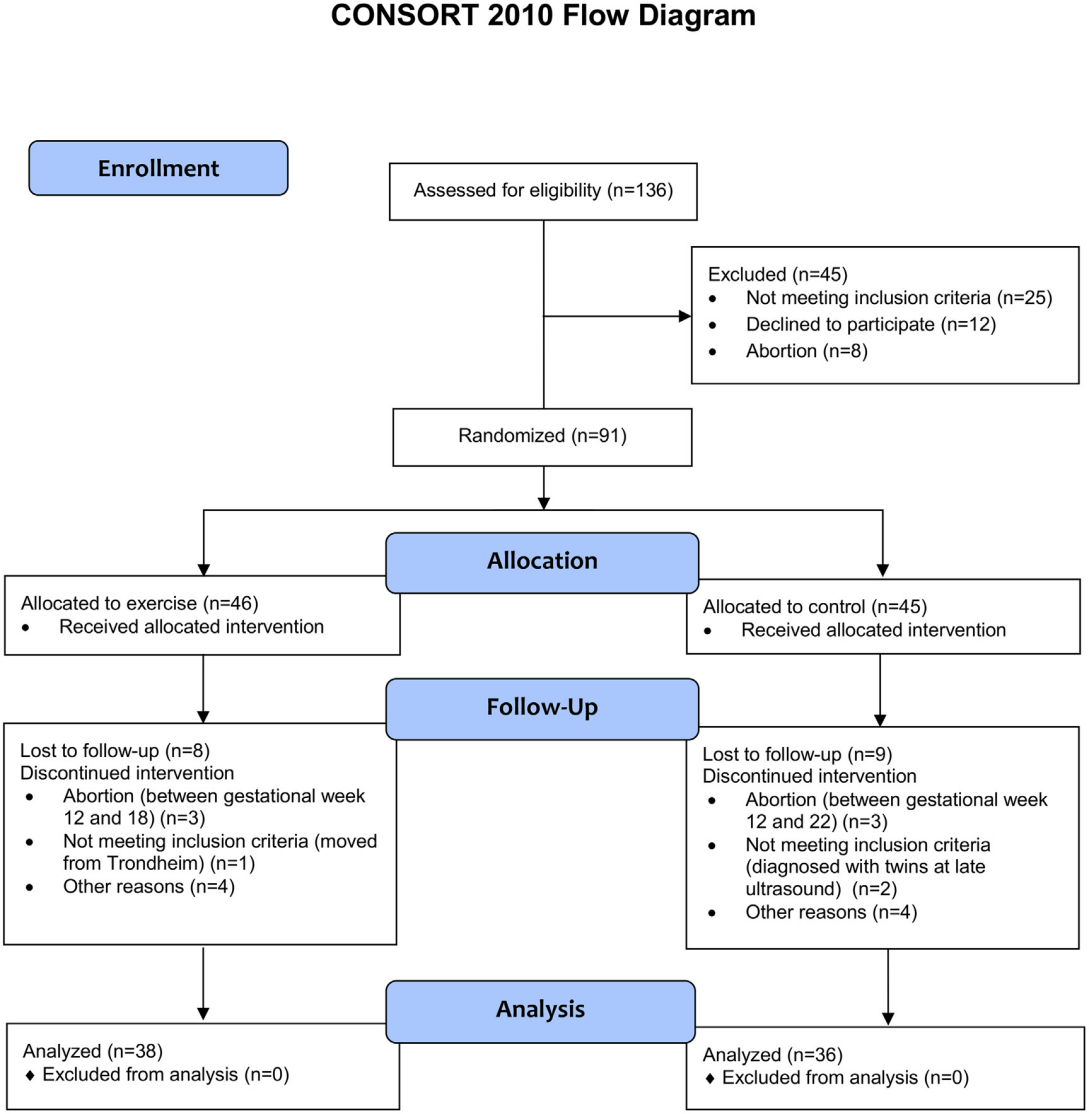
Flow chart of the ETIP study.

Fig 1 outlines the flow of participants during the trial.

Recruitment started on 20 September 2010 and was continued until 1 March 2015. The final data collection date for the primary outcome measure was 20 June 2015. The aim of our study was to include 150 participants, but enrollment was stopped on 1 March 2015 at 91 randomized participants, due to the prolonged time for inclusion and fewer eligible participants than expected. Table 1 shows the baseline characteristics of the participants.

**Table 1:** Baseline characteristics of all women included in the ETIP study.

Baseline Characteristic	Exercise Group (*n* = 46)	Control Group (*n* = 45)
**Age (years)**	31.3 ± 3.8	31.4 ± 4.7
**Weight (kg)**	95.3 ± 12.8	98.3 ± 14.2
**Height (cm)**	167.6 ± 5.9	167.1 ± 6.5
**BMI (kg/m** ^**2**^ **)**	33.9 ± 3.8	35.1 ± 4.6
**Weight classification**		
Overweight, BMI 28.0–29.9 kg/m^2^	3 (6.6%)	5 (11.1%)
Class 1 obesity, BMI 30.0–34.9 kg/m^2^	28 (62.2%)	19 (42.2%)
Class 2 obesity, BMI 35.0–39.9 kg/m^2^	11 (24.4%)	15 (33.3%)
Class 3 obesity, BMI ≥ 40.0 kg/m^2^	3 (6.6%)	6 (13.3%)
**Parity**		
0	22 (47.8%)	19 (42.2%)
1	19 (41.3%)	19 (42.2%)
2	5 (10.9%)	4 (8.9%)
≥3	0 (0.0%)	3 (6.7%)
**Current smoking**	2 (4.7%)	4 (8.9%)
**Education**		
Primary/secondary school	1 (2.3%)	3 (7.0%)
High school	15 (34.1%)	12 (27.9%)
University ≤4 y	14 (31.8%)	11 (25.6%)
University >4 y	14 (31.8%)	17 (39.5%)
**Currently employed**	38 (82.6%)	33 (73.3%)
**GDM**		
WHO 2009 definition*		
4 (8.7%)	4 (8.7%)	4 (8.9%)
WHO/IADPSG 2013 definition**		
8 (18.2%)	8 (18.2%)	13 (29.5%)
**Maternal hypertension**	3 (7.0%)	4 (9.5%)
**Body composition** ***		
Fat mass (kg)	40.0 ± 7.7	44.1 ± 10.3
Fat mass (percent)	43.1 ± 3.8	44.8 ± 5.5
Fat-free mass (kg)	52.4 ± 5.6	53.3 ± 6.1
Fat-free mass (percent)	56.9 ± 3.8	55.2 ± 5.5
**Skinfold thickness**		
Biceps area (mm)	20.3 ± 8.9	22.3 ± 8.8
Triceps area (mm)	28.8 ± 7.0	31.2 ± 7.2
Subscapular area (mm)	30.5 ± 8.6	33.1 ± 7.9
**Blood pressure**		
Systolic blood pressure (mm Hg)	126.3 ± 20.9	127.9 ± 12.9
Diastolic blood pressure (mm Hg)	75.0 ± 10.0	78.0 ± 8.4
**Blood measurements**		
Fasting glucose (mmol/l)	4.6 ± 0.4	5.0 ± 0.8
120-min glucose (mmol/l)	6.2 ± 1.1	6.1 ± 1.6
Insulin (pmol/l)	158.3 ± 62.5	150.0 ± 70.8
HbA1c (percent)	5.2 ± 0.3	5.4 ± 0.4
Insulin C-peptide (nmol/l)	0.7 ± 0.7	0.7 ± 0.4
Triglycerides (mmol/l)	1.4 ± 0.4	1.5 ± 0.2
Ferritin (pmol/l)	147.4 ± 97.5	84.9 ± 49.4
HDL cholesterol (mmol/l)	1.6 ± 0.3	1.8 ± 0.3
LDL cholesterol (mmol/l)	3.0 ± 0.9	3.0 ± 0.5
Total cholesterol (mmol/l)	5.0 ± 1.3	5.1 ± 1.2
Hemoglobin (g/l)	127.0 ± 100.0	126 ± 8.0
High-sensitivity CRP (mg/l)	8.4 ± 3.5	10.2 ± 5.0
HOMA2-IR	2.6 ± 1.2	2.5 ± 1.2

Observed data are presented as mean ± standard deviation or number of participants (percent). Missing: number of participants with missing data in each group is 0 to 3 for all variables except for body composition, where 14 are missing in the exercise group and 12 in the control group.

*Fasting plasma glucose ≥ 7.0 mmol/l or 120-min plasma glucose ≥ 7.8 mmol/l.

**Fasting plasma glucose ≥ 5.1 mmol/l or 120-min plasma glucose ≥ 8.5 mmol/l.

***Body composition was measured by air displacement plethysmography (BOD POD).

There were no significant differences between groups at baseline, except from mean fasting glucose (4.6 mmol/l in the exercise group, 5.0 mmol/l in the control group; *p* = 0.02). Table 2 shows the model-based analyses for the continuous primary and secondary outcomes. The mean number of weeks from inclusion to delivery was 23.3 (range 10–28) in the exercise group and 24.7 (range 19–30) in the control group. Mean gestational age was 39.5 wk (range 27–42 wk) in the exercise group and 39.4 wk (range 37–42) in the control group.

**Table 2:** Primary and secondary outcomes in late pregnancy and at delivery.

Outcome at Late Pregnancy/Delivery	Baseline Mean	Exercise Group (*n* = 38)		Control Group (*n* = 36)		Between-Group Comparison		
		Final Mean	95% CI	Final Mean	95% CI	Mean Difference	95% CI	*p*-Value
**GWG (kg) (primary outcome)**		10.5	8.9, 12.0	9.2	6.8, 11.6	1.29	−1.58, 4.05	0.35
**Weight (kg)**	96.8	107.1	103.9, 110.3	106.1	102.9, 109.3	0.92	−1.35, 3.18	0.43
**BMI(kg/m** ^**2**^ **)**	34.5	37.4	36.4, 38.4	37.0	36.1, 38.0	0.35	−0.45, 1.15	0.85
**Body composition** *								
Fat mass (kg)	42.2	46.4	43.8, 49.0	45.0	42.4, 47.7	1.35	−0.98, 3.68	0.26
Fat mass (%)	44.0	43.7	42.5, 45.0	43.3	42.0, 44.6	0.43	−0.67, 1.52	0.45
Fat-free mass (kg)	53.0	57.7	56.0, 59.4	58.3	56.6, 60.0	−0.59	−2.28, 1.11	0.50
Fat-free mass (%)	55.8	56.5	55.2, 57.8	56.5	55.2, 57.8	−0.05	−1.17, 1.07	0.93
**Skinfold thickness**								
Biceps area (mm)	21.3	18.5	16.2, 20.8	18.3	15.9, 20.6	0.23	−2.42, 2.89	0.86
Triceps area (mm)	30.0	28.0	26.1, 29.8	29.8	28.0, 31.6	−1.82	−3.82, 0.17	0.07
Subscapular area (mm)	31.8	30.8	28.6, 33.0	31.0	28.8−33.2	−0.26	−2.63, 2.12	0.83
**Blood pressure**								
Systolic BP (mm Hg)	124.5	120.4	116.4, 124.3	128.1	124.0, 132.2	−7.73	−13.23, −2.22	0.006
Diastolic BP (mm Hg)	76.0	76.6	73.8, 79.3	80.2	77.3, 83.0	−3.61	−7.42, 0.20	0.06
**Blood measurements**								
Fasting glucose (mmol/l)	4.7	4.6	4.4, 4.8	4.5	4.3, 4.7	0.09	−0.20, 0.37	0.56
120-min glucose (mmol/l)	6.0	6.2	5.6, 6.7	5.8	5.3, 6.4	0.33	−0.44, 1.10	0.40
Insulin (pmol/l)	142.4	209.0	179.9, 238.2	208.4	177.1, 238.9	0.9	−39.4, 41.1	0.97
HbA1c (%)	5.2	5.4	5.3, 5.5	5.4	5.3, 5.5	−0.06	−0.20, 0.08	0.41
Insulin C-peptide (nmol/l)	0.6	0.7	0.6, 0.9	0.8	0.7, 1.0	−0.10	−0.31, 0.11	0.37
Triglycerides (mmol/l)	1.4	2.6	2.3, 2.9	2.4	2.0, 2.7	−0.25	−0.77, 0.13	0.24
Ferritin (pmol/l)	127.0	29.7	7.0, 52.4	37.7	14.8, 60.9	−8.20	−40.18, 23.64	0.62
HDL cholesterol (mmol/l)	1.7	1.6	1.5, 1.7	1.7	1.6, 1.8	−0.08	−0.22, 0.06	0.27
LDL cholesterol (mmol/l)	2.8	3.6	3.3, 3.8	3.6	3.4, 3.9	−0.06	−0.43, 0.30	0.73
Total cholesterol (mmol/l)	5.0	6.1	5.8, 6.5	6.4	6.0, 6.8	−0.28	−0.81, 0.25	0.30
Hemoglobin (g/l)	127.0	118.0	115.0, 120.0	117.0	114.0, 120.0	1.1	−2.9, 5.1	0.59
High-sensitivity CRP (mg/l)	10.7	6.6	4.4, 8.8	6.5	4.3, 8.7	0.09	−3.01, 3.18	0.96
HOMA2-IR	2.5	3.6	3.2, 4.1	3.7	3.2, 4.2	−0.04	−0.68, 0.59	0.90

Intention-to-treat model-based analyses with baseline mean (all participants), mean and 95% CI at late pregnancy/delivery for the exercise group and the control group, and comparison between groups presented as mean difference, 95% CI, and *p*-value. The mother’s weight was measured and BMI calculated at delivery, the rest of the measurements were at gestational week 34–37. The number of participants with missing data in the exercise group varied between 0 and 5, in the control group between 0 and 3, for all variables except for body composition, where 12 participants in each group had missing data. The effect of treatment was assessed with linear mixed models. For the primary and secondary outcomes, the effect of time and treatment was taken as a fixed effect. Due to randomization, there were no systematic differences between groups at baseline. To account for repeated measurements, participant ID was included as a random effect.

*Body composition was measured by air displacement plethysmography (BOD POD).

BP, blood pressure.

### Gestational Weight Gain

We found no significant differences in GWG between the exercise group and the control group (Table 2). Body weight at delivery was self-reported by five women in the exercise group and four women in the control group. The proportion of women exceeding the IOM guidelines for recommended GWG was similar in the two groups (Table 3). Adjusting for gestational age in the analyses did not affect the GWG comparison between groups significantly (mean difference 0.56, *p* = 0.67).

**Table 3:** Secondary outcomes in late pregnancy and at delivery.

Outcome at Late Pregnancy/Delivery	Exercise Group (*n* = 38), *n* (Percent)	Control Group (*n* = 36), *n* (Percent)	Between-Group Comparison		
			Odds Ratio	95% CI	*p*-Value
**GDM**					
WHO 2009 definition*	2 (6.1)	9 (27.3)	0.1	0.016, 0.949	0.04
WHO/IADPSG 2013 definition**	5 (14.7)	8 (24.2)	0.5	0.126, 2.349	0.42
**Maternal hypertension**	3 (9.1)	7 (22.6)	0.2	0.019, 1.976	0.17
**GWG greater than IOM recommendations**	21 (58.3)	16 (44.4)	0.6	0.225, 1.453	0.35

Intention-to-treat analysis based on observed data for the exercise and the control group and comparison between groups are presented as number of participants (percentage), odds ratio, 95% CI, and *p*-value. The analyses of GDM and hypertension were done on the basis of blood tests and blood pressure measurements in late pregnancy. The analysis of GWG relative to IOM recommendations was done on the basis of weight measurements at delivery. The number of participants with missing data in the exercise group varied between 0 and 5, in the control group between 0 and 3. Data were analyzed by a mixed logistic regression model.

*Fasting plasma glucose ≥ 7.0 mmol/l or 120-min plasma glucose ≥ 7.8 mmol/l.

**Fasting plasma glucose ≥ 5.1 mmol/l or 120-min plasma glucose ≥ 8.5 mmol/l.

### Gestational Diabetes Mellitus

In late pregnancy, two women (6.1%) in the exercise group and nine women (27.3%) in the control group had developed GDM according to the WHO 2009 definition [27], with a statistical difference between groups (*p* = 0.04; Table 3). According to the WHO/IADPSG 2013 definition of GDM [28], there was no significant difference between the groups (Table 3). There was no significant difference in fasting glucose, 120-min glucose, insulin, or HbA1c level between the groups (Table 2).

### Blood Pressure and Other Secondary Outcomes

In late pregnancy we found a significantly lower systolic blood pressure (*p* = 0.006) in the exercise group compared to the control group (Table 2). There were no significant differences in other secondary outcome measures (Tables 2 and 3).

### Physical Activity

The proportion of women reporting to be physically active for at least 30 min each day in late pregnancy was equal in the two groups: 61% in the exercise group and 66% in the control group (*p* = 0.73). The proportion of women reporting regular exercise training in late pregnancy was significantly higher in the exercise than in the control group: 77% and 23%, respectively (*p* < 0.01).

### Per Protocol Analyses

In the exercise group, 50% of the women fulfilled the training intervention as described in the study protocol [23]. In the per protocol analyses, we found no significant difference in weight gain and mean weight at delivery between the per protocol exercise group and the control group (S1 Table). Resting systolic and diastolic blood pressure were significantly lower in the per protocol exercise group (115.7 mm Hg/75.1 mm Hg) compared to the control group (128.1 mm Hg/80.2 mm Hg), with *p* = 0.001 and *p* = 0.02, respectively. A tendency toward lower incidence of GDM (5.9% in the per protocol exercise group, 27.3% in the control group, *p* = 0.11) and maternal hypertension (11.1% in the per protocol exercise group, 21.2% in the control group, *p* = 0.14) was seen in the per protocol exercise group (S2 Table).

### Adverse Events

No adverse events were reported during the exercise training or study assessments (Table 4).

**Table 4:** Abortions, premature deliveries, and other adverse events occurring during follow-up.

Adverse Event	Exercise Group (*n* = 46)	Control Group (*n* = 45)
Abortion before gestational week 22	3 (6.5%)	3 (6.7%)
Delivery before gestational week 37	1 (2.2%)	1 (2.2%)
Other adverse events	0	0

Data are presented as number (percent).

## Discussion

### Main Findings

We found no difference in GWG between women randomized to an exercise training program versus standard maternity care, but found an apparent reduction in the incidence of GDM and lower systolic blood pressure in late pregnancy among the women randomized to the exercise training program. In the per protocol analyses including only the women who had adhered to the exercise program (*n* = 19), exercise training also seemed to reduce diastolic blood pressure in late pregnancy.

### Gestational Weight Gain

Our findings of no difference in GWG and body composition between groups are in line with several other clinical trials on overweight or obese pregnant women [14–16,32–34]. However, a systematic review by Sui and Dodd [20] that included 216 participants (five randomized trials) found that supervised exercise interventions were associated with lower GWG among overweight or obese pregnant women. But the trials included in this systematic review differed with respect to the type and duration of exercise, and a clinically relevant difference in weight gain was not precalculated. Also Barakat et al. [35] and Haakstad and Bø [36] found significantly lower GWG among women who participated in supervised exercise during pregnancy. However, these two studies included women from all weight classes, and their findings might not translate specifically to overweight/obese women. We can only speculate about why there was no difference in GWG between the two groups in the ETIP study. The proportion of women whose self-reported activity level fulfilled the recommended 30 min of daily physical activity in late pregnancy was higher in the exercise group, but some of the women in the control group exercised on their own. Only 50% of the women in the exercise group adhered to the exercise protocol as prescribed a priori [23]. A possible effect of regular exercise during pregnancy may have been missed in our study due to the relatively low adherence to the training protocol. Protocol adherence is a challenge in all exercise studies. We tried to improve adherence by offering motivational talks throughout the intervention period, as well as adjusting the training times so that more women would be able to attend. The low adherence may have been due to pregnancy symptoms such as tiredness and nausea, limited previous experience with exercise training, or difficulties in prioritizing time for exercise. Furthermore, the intervention protocol might have been too comprehensive for these women. Further studies should carefully consider how exercise adherence can be obtained in this population.

Although the exercise training program in our study followed the current recommendations for exercise in pregnancy, it is possible that the exercise frequency and/or intensity of our program were not sufficient to affect the outcome measures related to weight gain. As our study population had a relatively low fitness level, the amount of energy spent during the exercise sessions was rather low (~400 kcal/session) and was probably not sufficient to affect the energy balance significantly. It is also possible that some of the women in the exercise group compensated for energy expenditure during the exercise sessions either by decreasing their physical activity level during the remaining time of the week [37] or by increasing their energy intake [38]. According to three recent meta-analyses [32,39,40], interventions combining physical activity and diet have proven effective in reducing GWG in overweight and obese women. We did not include any dietary advice or intervention in our study, and probably exercise training alone is not sufficient to reduce GWG in this population.

Changes in body composition throughout pregnancy might be an important determinant of glucose metabolism. Few studies have assessed body composition changes after exercise in pregnancy. Our findings of no significant differences between groups in body composition in late pregnancy are in line with a recent RCT on the effects of a 16-wk moderate intensity cycling program in overweight and obese pregnant women [33].

### Gestational Diabetes Mellitus

Our finding of an apparently lower incidence of GDM according to the WHO 2009 definition [27] among the women in the exercise group is in line with a recent meta-analysis of 13 RCTs [22] that concluded that structured moderate intensity exercise programs during pregnancy decrease the risk of GDM. However, two previous Cochrane reviews, one on exercise as the sole intervention [19] and one on both diet and exercise interventions [41], concluded that there is no clear GDM risk reduction after exercise training. Nobles et al. [42] randomized 251 women with increased risk of GDM to either exercise training or a comparison health and wellness group and found no reduction in GDM risk after exercise, in line with another previous review [20]. The recently published DALI Lifestyle Pilot study [43] found that women with BMI ≥ 29 kg/m^2^ randomized to a healthy eating intervention had significantly lower fasting glucose and 2-h insulin concentrations than women in an exercise only group. In contrast to the DALI study, our results indicate that exercise training alone may be sufficient to prevent glucose intolerance in overweight or obese pregnant women. An important difference between the DALI study and ours is that the exercise training was supervised in our study.

Using the WHO/IADPSG 2013 definition [28] of GDM in the ETIP study, the number of GDM cases increased in both groups, and there was no longer a significant difference between the groups. The WHO/IADPSG 2013 definition is mainly based on the HAPO study (2008) [44], which found strong associations between glucose levels below the WHO 2009 diagnostic definitions and adverse outcomes for both mother and child. However, a retrospective cohort study [45] that included 1,892 women diagnosed with GDM according to the WHO/IADPSG 2013 definition found a significantly higher risk for adverse pregnancy outcomes in those who also would be diagnosed as having GDM according to the WHO 2009 definition.

Despite the difference between the exercise and control groups in GDM incidence, we found no differences between the groups at late pregnancy in glucose levels, insulin, or HOMA2-IR. One possible reason for this finding is that women with high risk of GDM may respond differently to exercise training than women with lower risk [46], such that the average glucose and insulin levels are not sufficiently affected to obtain a difference between groups.

### Blood Pressure

We found a significantly lower systolic blood pressure among the women in the exercise group in late pregnancy, compared to the women in the control group. Diastolic blood pressure did not differ between groups in late pregnancy in the intention-to-treat analysis, but was significantly lower in the exercise group in the per protocol analysis. High blood pressure in pregnancy is associated with increased risk for preeclampsia [47] and thus is important to prevent. To our knowledge, only one previous RCT [33] has studied the effect of exercise training in pregnancy on exact blood pressure measurements among overweight/obese women. Seneviratne et al. [33] found no effect of exercise training on blood pressure in late pregnancy. Other studies that have assessed the effects of exercise on maternal hypertension risk have assessed hypertension as a dichotomous variable [34,35,39]. Some of these studies found no effect of exercise [17,34], but one study [35] found a reduced incidence of maternal hypertension after exercise training. The latter study included both normal weight, overweight, and obese women. Although fewer women in the exercise group than in the control group had hypertension in late pregnancy in our study, the difference was not statistically significant. Further studies are needed to ascertain whether exercise training can prevent hypertensive pregnancies in overweight/obese women.

### Generalizability

The ETIP study had few exclusion criteria, and the participants were representative of Norwegian women with BMI ≥ 28 kg/m^2^ regarding age, parity, and education. However, participants had to have time available for the testing and training. The exercise group was offered training sessions at day and evening times. It is also possible that the participants volunteering for the ETIP study were extra aware of the possible beneficial effects of exercise training in pregnancy and thus were motivated to participate in our trial.

### Clinical Relevance

Obese women have elevated risk of GDM and maternal hypertension; thus, finding effective prevention strategies is highly relevant. The study revealed no adverse events related to moderate physical activity during pregnancy. The effect of exercise training to reduce weight gain may most likely be improved with additional dietary interventions. During the study we experienced difficulties in motivating the women in the exercise group to adhere to the training program, despite supervised training sessions at St. Olavs Hospital, training sessions at different times during the week, and individually adjusted exercises. We think further studies should evaluate how supervised exercise programs for obese women can be implemented in the health care system, as well as how to obtain good adherence to such programs.

### Strengths

In our study, exercise training was the only intervention provided. This makes it easier to assess the isolated effects of exercise on pregnancy outcomes. The training program being standardized and supervised makes it easy to reproduce. Furthermore, we had thorough recording of exercise adherence as well as physical activity levels in the two groups. The primary outcome measure (GWG) was assessed by personnel blinded for group allocation. We also regard the assessment of body composition with the gold standard method of air displacement plethysmography as a strength.

### Limitations

The main limitation of the trial was the reduced statistical power because we were able to include only 2/3 of the 150 participants estimated in the power calculation. We analyzed 30 different secondary outcomes among a limited number of women, and thereby increased the risk for detecting differences between groups by chance, and making type 1 errors. Furthermore, only 50% of the participants in the exercise group performed the exercise training program per protocol, which makes it more difficult to detect possible effects of the intervention. However, adherence to exercise in the ETIP study was similar to that in most of the comparable clinical studies. Care must be taken in interpreting the results from the per protocol analysis. Such analyses could be selection biased if the reasons influencing compliance with the exercise training program are associated with prognostic factors [48].

### Conclusion

In this trial we did not observe a reduction of GWG or an improvement in body composition among overweight/obese women who were offered supervised exercise training during pregnancy. However, exercise training seemed to reduce the incidence of GDM as well as systolic blood pressure in late pregnancy. As exercise adherence is a major challenge in this population, there is a special need to find methods to reduce participant attrition in future studies.

## Supporting Information

S1 Data Participant characteristics and outcomes in late pregnancy. (SAV)

S2 Data Physical activity in early pregnancy. Part of a questionnaire.
(SAV)

S3 Data Physical activity in late pregnancy. Part of a questionnaire.
(SAV)

S1 Table Outcome measurements comparing participants in the exercise group who adhered to the training protocol with the control group. Supplementary material, per protoco1. Secondary outcomes in late pregnancy and at delivery.
(DOCX)

S2 Table Outcome measurements comparing participants in the exercise group who adhered to the training protocol with the control group—binary data. Supplementary material, per protoco1. Secondary outcomes in late pregnancy and at delivery.
(DOCX)

S1 Text Trial protocol. (DOCX)

S2 Text CONSORT statement. (DOC)

S3 Text Information given to the participants in the ETIP study. (DOCX)

S4 Text Information given to the participants in the exercise group. (DOC)

S5 Text Exercise recording form for training sessions at St. Olavs Hospital. (DOC)

S6 Text Exercise program for training sessions at St. Olavs Hospital. (DOC)

S7 Text Exercise recording from for training and physical activity at home. (PDF)

S8 Text Home-based exercise program. (DOC)

S9 Text Questionnaire regarding general physical activity at baseline. (PDF)

S10 Text Questionnaire regarding general physical activity at late pregnancy. (PDF)

S11 Text Funding: the Norwegian Fund for Post-Graduate Training in Physiotherapy. (DOCX)

S12 Text Funding: the Liaison Committee between the Central Norway Regional Health Authority (RHA) and the Norwegian University of Science and Technology (NTNU). (PDF)

S13 Text Biobank approval. (DOC)

S14 Text The Regional Committee for Medical and Health Research Ethics (REK midt) approval. (PDF)

## Abbreviations

BMI: body mass index
CRP: C-reactive protein
ETIP: Exercise Training in Pregnancy
GDM: gestational diabetes mellitus
GWG: gestational weight gain
HDP: high-density lipoprotein
IADPSG: International Association of Diabetes and Pregnancy Study Groups
IOM: Institute of Medicine
LDL: low-density lipoprotein
NTNU: Norwegian University of Science and Technology
RCT: randomized controlled trials
