# Supplementary material for: Exercise Training and Weight Gain in Obese Pregnant Women: A Randomized Controlled Trial (ETIP Trial)
Source: PLoS Med. 2016 Jul 26;13(7):e1002079. doi: 10.1371/journal.pmed.1002079 (PMC4961392; doi:10.1371/journal.pmed.1002079)
Supplement: S1 Table — Supplementary material, per protoco1. Secondary outcomes in late pregnancy and at delivery. (DOCX) [file pmed.1002079.s004.docx]

*S1 Table. Supplementary material, per protoco1. Primary and secondary outcomes in late pregnancy and at delivery. “Per protocol” model based analyses with baseline mean (all participants, final mean and 95% CI for the “per protocol” group and the control group, and comparison between groups presented by mean difference, 95% CI and p-value. Weight is calculated at delivery, the rest of the measurements are at gestational week 34-37.*

|  |  | Per protocol  Exercise group  (n = 19) | | Control group  (n = 36) | | Between-groups comparisons | | |
| --- | --- | --- | --- | --- | --- | --- | --- | --- |
|  | *Baseline*  *mean* | *Final*  *mean* | *95% CI* | *Final*  *mean* | *95% CI* | *Mean diff* | *95% CI* | *P* |
| *Primary* |  |  |  |  |  |  |  |  |
| Weight (kg) | 96.3 | 106.2 | 102.2, 110.2 | 105.7 | 102.1, 109.4 | 0.52 | -2.38, 3.42 | 0.73 |
| Weight gain (kg) |  | 9.9 | 7.5, 12.3 | 9.4 | 7.7, 11.1 |  |  |  |
|  |  |  |  |  |  |  |  |  |
| *Secondary* |  |  |  |  |  |  |  |  |
| Body mass index (BMI) | 34.6 | 37.4 | 36.1, 38.6 | 37.1 | 36.0, 38.2 | 0.28 | -0.73, 1.29 | 0.59 |
| **Body composition*** |  |  |  |  |  |  |  |  |
| Fat mass kg | 42.0 | 46.1 | 42.7, 49.5 | 44.9 | 41.9, 47.8 | 1.25 | -1.80, 4.30 | 0.42 |
| Fat mass % | 44.1 | 43.0 | 41.4, 44.6 | 43.3 | 41.9, 44.8 | -0.35 | -1.64, 0.94 | 0.60 |
| Fat free mass kg | 52.5 | 58.5 | 56.4, 60.6 | 57.8 | 56.1, 59.6 | 0.65 | -1.18, 2.48 | 0.49 |
| Fat free mass % | 55.9 | 57.0 | 55.4, 58.6 | 56.7 | 55.2, 58.1 | 0.35 | -0.94, 1.64 | 0.60 |
| **Skinfold thickness:** |  |  |  |  |  |  |  |  |
| Biceps area  Triceps area  Scapulae area | 21.5  30.0  32.1 | 18.4  28.8  30.6 | 15.2, 21.5  26.4, 31.3  27.7, 33.6 | 18.4  29.8  31.3 | 15.8, 20.9  27.8, 31.9  28.9, 33.7 | 0.02  -1.00  -0.64 | -3.32, 3.37  -3.47, 1.47  -3.70, 2.42 | 0.99  0.43  0.68 |
| **Blood pressure (BP)** |  |  |  |  |  |  |  |  |
| Systolic BP (mmHg) | 124.4 | 115.7 | 110.0, 121.5 | 128.1 | 123.6, 132.5 | -12.4 | -19.44, -5.25 | 0.001 |
| Diastolic BP (mmHg) | 76.0 | 75.1 | 71.6, 78.7 | 802 | 77.4, 82.9 | -5.1 | -9.39, -0.72 | 0.02 |
| **Blood measurements** |  |  |  |  |  |  |  |  |
| Fasting glucose (mmol/L) | 4.8 | 4.5 | 4.2, 4.8 | 4.5 | 4.3, 4.8 | -0.04 | -0.42, 0.34 | 0.83 |
| 120 min glucose (mmol/L) | 6.0 | 6.3 | 5.5, 7.1 | 5.8 | 5.3, 6.4 | 0.48 | -0.50, 1.46 | 0.33 |
| Insulin (pmol/L) | 136.8 | 200.7 | 163.9, 237.5 | 205.6 | 177.1, 234.7 | -4.9 | -50.6, 40.8 | 0.83 |
| HBA1c (%) | 5.2 | 5.4 | 5.3, 5.5 | 5.4 | 5.3, 5.5 | 0.03 | -0.21, 0.14 | 0.72 |
| Insulin C-peptide (nmol/L) | 0.6 | 0.9 | 0.7, 1.1 | 0.8 | 0.7, 1.0 | 0.04 | -0.18, 0.27 | 0.71 |
| Triglycerides (mmol/L) | 1.4 | 2.8 | 2.4, 3.2 | 2.4 | 2.0, 2.7 | 0.41 | 0.13, 0.96 | 0.13 |
| Ferritin (pmol/L) | 117.3 | 25.8 | 0.45, 51.23 | 34.6 | 16.0, 53.3 | -8.70 | -40.20, 22.80 | 0.59 |
| HDL cholesterol (mmol/L) | 1.7 | 1.6 | 1.5, 1.8 | 1.7 | 1.6, 1.8 | -0.09 | -0.25, 0.07 | 0.29 |
| LDL cholesterol (mmol/L) | 2.9 | 3.5 | 3.1, 3.9 | 3.6 | 3.4, 3.9 | -0.14 | -0.58, 0.29 | 0.52 |
| Cholesterol (mmol/L) | 5.0 | 5.8 | 5.3, 6.3 | 6.4 | 6.0, 6.8 | -0.64 | -1.28, 0.00 | 0.05 |
| Hemoglobin (g/L) | 127.0 | 117.0 | 113.0, 120.0 | 117.0 | 114.0, 120.0 | -0.1 | -4.2, 4.3 | 0.97 |
| High sensitive CRP (mg/L) | 10.7 | 6.2 | 3.1, 9.4 | 6.5 | 4.2, 8.8 | -0.29 | -4.15, 3.57 | 0.88 |
| HOMA2-IR | 2.4 | 3.5 | 2.9, 4.1 | 3.6 | 3.2, 4.1 | -0.19 | -0.92, 0.53 | 0.60 |
|  |  |  |  |  |  |  |  |  |
| *Missing: Number of missing in the “per protocol” group varies between 0 and 5, in the control group between 0 and 3, except for body composition where there were 6 missing in the exercise group and 12 in the control group.*  *Statistics: The effect of treatment was assessed with linear mixed models. For the primary and secondary outcomes, the effect of time and treatment was taken as a fixed effect. Due to randomization, there are no systematic differences between groups at baseline. To account for repeated measurements, participant ID was included as a random effect.*  *Abbreviations: HDL: High density lipoprotein, LDL: Low density lipoprotein, HBA1c: Glycated hemoglobin, CRP: C-reactive protein, HOMA2IR: Homeostatic Model Assessment of insulin resistance.*  ** Body composition was measured by air displacement plethysmography (BOD POD).* | | | | | | | | |
