# Supplementary material for: Exercise Training and Weight Gain in Obese Pregnant Women: A Randomized Controlled Trial (ETIP Trial)
Source: PLoS Med. 2016 Jul 26;13(7):e1002079. doi: 10.1371/journal.pmed.1002079 (PMC4961392; doi:10.1371/journal.pmed.1002079)
Supplement: S2 Table — Supplementary material, per protoco1. Secondary outcomes in late pregnancy and at delivery. (DOCX) [file pmed.1002079.s005.docx]

*S2 Table. Supplementary material, per protoco1. Secondary outcomes in late pregnancy and at delivery. “Per protocol” analysis based on observed data for the per protocol exercise and the control group and comparison between groups are presented in number of participants (N), percentage (%),odd ratio (OR), 95% confidence interval (CI), and p-value. Analyses of gestational diabetes mellitus and hypertension were done on basis of blood tests and blood pressure measurements at late pregnancy. Analyses of weight gain according to IOM recommendations were* *done one basis of weight measurements at delivery.*

| **Outcomes**  **late pregnancy/delivery** | **Per protocol**  **Exercise group**  **N = 19** | **Control group**  **N = 36** | **Between groups comparisons** | | |
| --- | --- | --- | --- | --- | --- |
|  | *n (%)* | *n (%)* | *Odds Ratio* | *95% CI* | *P-value* |
|  |  |  |  |  |  |
| Gestational diabetes mellitus |  |  |  |  |  |
| WHO 2009* | 1 (5.9) | 9 (27.3) | 0.1 | 0.007, 1.675 | 0.11 |
| WHO/IADPSG 2013** | 2 (13.9) | 8 (27.3) | 0.4 | 0.057, 2.597 | 0.33 |
| Maternal hypertension | 1 (11.1) | 7 (21.2) | 0.8 | 0.003, 2.294 | 0.14 |
| >IOM recommendations | 11 (57.9) | 16 (44.4) | 1.7 | 0.559, 1.529 | 0.40 |
|  |  |  |  |  |  |
| ***Missing:*** *Number of missing in the exercise varies between 0 and 2, in the control group between 0 and 3.*  ***Statistics:*** *Data were analyzed by Mixed logistic regression.*  ***Abbreviations:*** *WHO: World Health Organization. IADPSG: The International Association of the Diabetes and Pregnancy Study Groups, IOM: The Institute of Medicine recommendations for weight gain during pregnancy for overweight or obese women.*  ****Definition:*** *Fasting plasma glucose ≥ 7.0 mmol/L, or 2 h concentration ≥ 7.8 mmol/L.*  *****Definition:*** *Fasting plasma glucose ≥ 5.1 mmol/L, or 2 h ≥ 8.5 mmol/L.* | | | | | |
