# Supplementary material for: Exercise Training and Weight Gain in Obese Pregnant Women: A Randomized Controlled Trial (ETIP Trial)
Source: PLoS Med. 2016 Jul 26;13(7):e1002079. doi: 10.1371/journal.pmed.1002079 (PMC4961392; doi:10.1371/journal.pmed.1002079)
Supplement: S1 Text — (DOCX) [file pmed.1002079.s006.docx]

# Exercise Training in Pregnancy for overweight and obese women

**Summary**

Being overweight or obese is associated with several adverse pregnancy outcomes. Exercise training is recommended for all pregnant women, and is possibly extra important for overweight and obese women. There is a lack of good quality randomized controlled trials on the effects of regular exercise training in pregnancy for overweight and obese women. We will assess if women with body mass index at or above 28 kg/m^2^ who exercise throughout pregnancy will have reduced gestational weight gain, reduced prevalence of gestational diabetes mellitus and improved insulin sensitivity compared to control women. The Exercise Training in Pregnancy for obese women (ETIP) study [[1](#_ENREF_1)] was started in 2010 and has now recruited almost 50 women. We apply for a PhD candidate to finish the data collection in the study and to write about the main findings on gestational weight gain, prevalence of gestational diabetes and insulin sensitivity.

**Background and status of knowledge**

In the Unites States two out of three reproductive age women are overweight (Body Mass Index (BMI) 25.0-29.9 kg/m^2^) or obese (BMI ≥ 30 kg/m^2^) [[2](#_ENREF_2)]. Obesity is associated with a number of adverse pregnancy outcomes, such as gestational diabetes, preeclampsia, caesarean delivery and children born large for gestational age [[3](#_ENREF_3)]. Over 60% of overweight women gain more weight than recommended during pregnancy [[4](#_ENREF_4)]. Such excess gestational weight gain could accelerate the obesity epidemic as it is directly associated with maternal weight retained during the postpartum period [[4](#_ENREF_4)] as well as with offspring adiposity in childhood [[5](#_ENREF_5)] and in early adulthood [[6](#_ENREF_6)]. Weight increase > 15 kg implies a higher risk of developing diseases in pregnancy and complications during labour [[7](#_ENREF_7)], as well as problems with weight retention after delivery [[8](#_ENREF_8)].

Gestational diabetes is defined as carbohydrate intolerance with onset or first recognition during pregnancy [[9](#_ENREF_9)], and is a risk factor for pregnancy complications [[10](#_ENREF_10)]. Women with gestational diabetes are at increased risk for developing type 2 diabetes later in life [[11](#_ENREF_11)]. It has been seen from observational studies that physical inactivity is associated with increased risk of gestational diabetes [[12](#_ENREF_12)]. In a recent study, it was found that women with normal body mass index randomized to an exercise program during pregnancy did not differ in prevalence of gestational diabetes compared to a control group [[13](#_ENREF_13)]. However, in this study, only 55% of the women managed to follow the recommended exercise protocol.

Pregnant women are currently advised to exercise with moderate intensity for 30 minutes or more on most, if not all, days of the week [[14](#_ENREF_14)]. Most women are not active enough during pregnancy and women who have a high pre-pregnancy BMI are even less likely to be physically active [[15](#_ENREF_15)]. Current knowledge about the effects of exercise on weight gain in pregnancy is mainly based on results from observational studies [[5](#_ENREF_5), [16-18](#_ENREF_16)]. Previous randomized trials (RCT) of physical activity during pregnancy are few in number and have had varying results. Some has found significantly less weight gain in women randomized to exercising in pregnancy [[19](#_ENREF_19)], while others have found no significant effects of exercise on gestational weight gain [[20-22](#_ENREF_20)]. There are several possible reasons for the divergent results; among these are low compliance, inadequately powered studies, and inadequate amount of exercise for the intervention groups. There is a lack of good quality RCTs assessing the effects on gestational weight gain, gestational diabetes prevalence, insulin resistance, and pregnancy complications of regular exercise in pregnancy. In particular, there is a need for studies on overweight and obese women as these constitute a high risk population.

**Aims of the study**

The main aim of the study is to assess if regular exercise in pregnancy can reduce weight gain in women with a pre-pregnancy BMI of 28 kg/m^2^ or more. We wish to test the hypothesis that overweight/obese women who exercise in addition to usual pregnancy care will have a lower gestational weigh gain compared to women who receive usual care only. Secondary aims include assessing if regular exercise in pregnancy can prevent or influence

- post partum weight retention
- body composition in mother and newborn
- insulin resistance/sensitivity
- prevalence of gestational diabetes
- lumbopelvic pain
- urinary and/or fecal incontinence
- prolonged labor and other complications
- endothelial function
- oxygen uptake
- psychological wellbeing
- impaired cardiac function in mother and fetus/newborn

The focus of this particular application is the primary outcome measure of gestational weight gain, as well as postpartum weight retention, insulin resistance and prevalence of gestational diabetes, body composition and objectively measured physical activity during pregnancy and postpartum. We will get data to four papers as outlined below:

1. The effect of exercise in pregnancy on gestational weight gain. This will be the main paper in the PhD. This paper includes the primary outcome of the study and we will test the hypothesis of reduced gestational weight gain in overweight/obese women who exercise compared to the control group. This study also includes changes in body composition, physical activity, and oxygen uptake from baseline to late pregnancy.
2. The effect of exercise in pregnancy on postpartum weight retention and body composition. This will be a follow-up paper to paper 1; we will assess the women and their babies 3 months postpartum to see if regular exercise during pregnancy has long term effects on weight and body composition.
3. Changes in levels of physical activity and body composition throughout pregnancy in overweight and obese women. We will assess if participation in the exercise training group prevents a drop in physical activity in late pregnancy.
4. The effects of exercise on insulin resistance/sensitivity. We will investigate the effects on the prevalence of gestational diabetes and insulin sensitivity of exercise training during pregnancy.

**Methods**

***Participants and setting***

Women with a self-reported pre-pregnancy BMI of 28 kg/m^2^ or more will be invited to participate. They have to be 18 years or older with a singleton live fetus at an 11-14 week ultrasound scan. Exclusion criteria includes high risk for preterm labour or diseases that could interfere with participation, and habitual exercise training (twice or more weekly) in the period before inclusion. The women are recruited through general practitioners and midwives, and also through invitations sent from the outpatient clinic for pregnant women at the St. Olav’s hospital. Tests and exercise training in the trial will be done at the Norwegian University of Science and Technology and the St. Olav’s hospital, Trondheim University Hospital. The recruitment started in September 210 and will continue until the needed number of participants is reach, anticipated until the end of January 2015.

***Intervention***

Standard dietary advice is given to all women. UThe training groupU follows a specially designed exercise program including aerobic activity, specific exercises for stabilization of the lower back and pelvis, and the pelvic floor muscles. They attend training groups for a total of 60 minutes 2 (-4) times weekly between 14 and 34 pregnancy weeks. The women are also encouraged to come for exercise training in the weeks after week 34 if they feel like. All women will have to come to exercise training for a minimum of 2 times weekly, and have the opportunity to attend as much as 4 times per week if they like. The endurance training consists of walking on treadmills for 25 minutes after warming up in 10 minutes. The intensity will be moderate, reaching ~80% of their maximal capacity in periods (corresponding to Borg scale12-15) [[23](#_ENREF_23)]. In addition, they follow a 50 minutes home exercise program at least once per week (35 minutes endurance training and 15 minutes strength exercises) as well as doing daily pelvic floor muscle exercises. They are also recommended to be physically active in addition to the training program. Adherence is strongly emphasized and registered in the women's personal training diary and the reports from the persons leading the training groups. The training protocol follows recommendations from The Norwegian Health Directory [[24](#_ENREF_24)] and the American College of Obstetrics and Gynecology [[14](#_ENREF_14)]. Specific adjustments are made according to aerobic activities if needed (for example using a stationary bike instead of treadmill walking). During the training period, the subjects will go through motivational interviews in a group setting. UWomen in the control group will receive the customary regular consultations with midwife, general practitioner and obstetrician. They are not discouraged from exercising on their own.

***Outcome measures***

*(At 14 and 34 weeks of pregnancy and 3 months postpartum)*

Primary outcome measure is weight increase based on weight measured at 14 weeks and immediately before labour. Maternal body weight will be measured by a calibrated electronic scale (SECA 770, Medema, Norway) to the nearest 0.1 kg. The participant will be wearing indoor clothing, without shoes. Weight at the point of delivery will be measured by hospital staff [[1](#_ENREF_1)].

Secondary outcome measures in this application are:

- Weight, Body Mass Index (BMI), skin-fold thickness (subscapular and triceps skinfold thickness measurements by Harpenden Caliper, sum of subscapular and triceps skinfold thicknesses and the ratio between these; S-T- ratio). Waist circumference is measured at all time-points at the level of the umbilicus.
- Body composition will be measured using air plethysmography (BodPod) at all three time points.
- Physical activity will be measured with SenseWear Armband activity monitors as well as through questionnaire.
- Gestational diabetes prevalence. Gestational diabetes will be measured by blood sampling for glucose concentrations taken after a 10 hour overnight fast. Glucose tolerance will be measured by a 2 hour 75 mg per oral test. A homeostasis model assessment (HOMA-IR) will be used to calculate insulin resistance. Gestational diabetes mellitus (GDM) is defined as fasting glucose ≥ 6,9 mmol/L or 2 h concentration ≥ 7,8 mmol/L [[25](#_ENREF_25)]

***Power calculation***

The power calculation has taken into account a 6 kg expected and clinical relevant difference between mean weight increases in the control group compared to the training group (between 14 weeks of pregnancy and labour) [[26](#_ENREF_26), [27](#_ENREF_27)]. Based on this assumption an independent samples t-test, 5% level of significance and test strength of 0.90 give a study population of 59 in each group. A 15% estimated drop-out requires a total of 150 included overweight/obese pregnant women.

***Statistical analyses***

The principal analysis will be done on an intention-to-treat basis; outcome measures will be analysed according to the treatment arm to which the subjects were randomised regardless of subsequent crossover or non-adherence. To model the outcome variables over time, we will use a linear mixed model with age, parity, and BMI as potential covariates to improve precision [[28](#_ENREF_28)]. In addition to this, we will split the women according to if they have actually exercised or not. The cut-off for this will be: 1) attending ≥ 42 organised training sessions, or 2) attending ≥ 28 organised sessions + performing ≥ 28 home exercise sessions, or 3) performing ≥ 60 home exercise sessions. To count as a home exercise session, they should exercise for at least 50 minutes with at least moderate intensity. We also will compare the women who are fulfilling the general recommendations for healthy adults of moderate intensity exercise for 30 minutes daily [[29](#_ENREF_29)] with women who are not fulfilling this. Results will be given as mean values with 95% confidence intervals (CI). P-values < 0.05 will be considered significant.

***Ethical considerations***

The Regional Committee for Medical Research Ethics has approved the study. The procedures followed will be in accordance with the Helsinki declaration. All participants will give their informed, written consent to participate.

**Main activities and milestones in the project period**

| **01.01.13 – 31.12.15** | **2013**  **1 2 3 4** | | | | **2014**  **1 2 3 4** | | | | **2015**  **1 2 3 4** | | | |
| --- | --- | --- | --- | --- | --- | --- | --- | --- | --- | --- | --- | --- |
| Inclusion/intervention | x | x | x | x | x | x | x | x | x |  |  |  |
| Data collection, statistics |  | x | x | x | x | x | x | x | x | x | x |  |
| Writing of article 1 |  |  |  |  |  |  |  |  | x | x |  |  |
| Writing of article 2 |  |  |  |  |  |  |  |  |  | x | x |  |
| Writing of article 3 |  |  |  |  |  |  |  |  |  |  | x | x |
| Writing of article 4 |  |  |  |  |  |  |  |  |  |  | x | x |
| Writing of PhD thesis |  |  |  |  |  |  |  |  |  |  | x | x |

**Tentative titles of the articles in the PhD**

1. Reduced weight gain in overweight and obese women who exercise during pregnancy. A randomised controlled trial.
2. Does exercise during pregnancy in overweight/obese women prevent postpartum weight retention? Results from a randomised controlled trial.
3. Changes in levels of physical activity and body composition throughout pregnancy in overweight and obese women. The effects of participating in a regular exercise training group.
4. Regular exercise training during pregnancy prevents gestational diabetes mellitus in overweight and obese women. A randomized controlled trial.

Tentative title of the PhD thesis will be “Exercise training in pregnancy for overweight and obese women.”

**Project management, organization and cooperation**

The project managers have broad experience in physiotherapy, exercise physiology, obstetrics and gynaecology, as well as experience in working with data from large clinical trials and population based studies. Supervisors of the PhD candidate Kirsti Krohn Garnæs will be Siv Mørkved and Trine Moholdt. ***Siv Mørkved*** is a physiotherapist and professor at the Faculty of Medicine, NTNU. Her main field of research has been in women’s health. Most of her publications are related to effects of interventions to treat pelvic floor dysfunction and pregnancy-related diseases. Dr. Mørkved has initiated and been the project manager of a number of RCTs in different patient groups at St. Olav University Hospital. She is principal mentor for three PhD candidates and co-mentor for two. ***Trine Moholdt*** is a physiotherapist and post doc at the Faculty of Medicine, NTNU. Her main field of research is exercise physiology. Her thesis was on the effects of aerob exercise training in coronary heart disease. She has been the project manager of several RCTs with exercise training as the intervention. ***Kjell Å. Salvesen*** is professor of obstetrics and gynecology at NTNU. He is consultant at the National Center for Fetal Medicine, St. Olav University Hospital, and vice-chair at the Department of Laboratory Medicine, Women’s and Children’s Health. His main field of research has been within fetal medicine, safety aspects of ultrasound in pregnancy, pre-eclampsia, post-term births and physical activity in pregnancy. He has published more than 80 papers, 40 of these during the last 5 years. He has been principal or co-mentor of seven PhD candidates. He is currently mentor of five other candidates.

***Torstein Vik*** is professor of paediatrics and perinatal epidemiology. His thesis was on long term consequences of IUGR, in particular of growth, mental and motor development as well as on physical health. This study was population based multicenter sponsored by the National Institute of Child Health and Development, Bethesda, MD, USA. Vik has also been significantly involved in long term neuro- psychiatric consequences of children, adolescents and young adults who had low birth weight. Vik has published more than 80 papers on various pediatric topics, 25 of these during the last five years. In a number of papers dr. Vik has also addressed the early origin of adult disease hypothesis. He has been mentor of four PhD candidates, and is currently mentor of six other candidates.

Other participants in our research group are professor **Pål Romundstad** (statistics), professor **Ulrik Wisløff** and researcher **Charlotte Björk Ingul** at the K.G.Jebsen Center of Exercise in Medicine.

***National collaboration*** is established with PT and professor in exercise physiology **Kari Bø**, Norwegian School of Sport Sciences and Akershus University Hospital. Professor Bø is an internationally highly respected researcher and opinion leader in women’s health, and has worked with Mørkved in several projects.

***International collaboration*** has already been established with a leading research group at Harvard Medical School, Boston, USA. **Dr. Emily Oken** is an assistant professor in the Department of Ambulatory Care and Prevention at Harvard Medical School and Harvard Pilgrim Health Care. She has clinical training in both Internal Medicine and Pediatrics. She has extensive experience in performing observational studies regarding associations of maternal diet, physical activity, and other behaviours with both maternal and infant health. For example, she has studied associations of maternal gestational weight gain with child obesity risk, diet during pregnancy with risk for gestational diabetes, physical activity before and during pregnancy with risk for gestational diabetes mellitus, and maternal diet and physical activity with postpartum weight retention. Dr. Emily Oken has visited us in Trondheim and has been an active part in the design of the study protocol.

**References**

1. Moholdt, TT, Salvesen, K, Ingul, CB, Vik, T, Oken, E ,Morkved, S: (2011) Exercise Training in Pregnancy for obese women (ETIP): study protocol for a randomised controlled trial. Trials, **12**:154.

2. Flegal, KM, Carroll, MD, Ogden, CL ,Curtin, LR: (2010) Prevalence and trends in obesity among US adults, 1999-2008. JAMA, **303**:235-41.

3. Baeten, JM, Bukusi, EA ,Lambe, M: (2001) Pregnancy complications and outcomes among overweight and obese nulliparous women. Am J Public Health, **91**:436-40.

4. Walker, LO, Sterling, BS ,Timmerman, GM: (2005) Retention of pregnancy-related weight in the early postpartum period: implications for women's health services. J Obstet Gynecol Neonatal Nurs, **34**:418-27.

5. Oken, E, Taveras, EM, Kleinman, KP, Rich-Edwards, JW ,Gillman, MW: (2007) Gestational weight gain and child adiposity at age 3 years. Am J Obstet Gynecol, **196**:322 e1-8.

6. Mamun, AA, O'Callaghan, M, Callaway, L, Williams, G, Najman, J ,Lawlor, DA: (2009) Associations of gestational weight gain with offspring body mass index and blood pressure at 21 years of age: evidence from a birth cohort study. Circulation, **119**:1720-7.

7. Clapp, JF, 3rd ,Little, KD: (1995) Effect of recreational exercise on pregnancy weight gain and subcutaneous fat deposition. Med Sci Sports Exerc, **27**:170-7.

8. Oken, E, Taveras, EM, Popoola, FA, Rich-Edwards, JW ,Gillman, MW: (2007) Television, walking, and diet: associations with postpartum weight retention. Am J Prev Med, **32**:305-11.

9. (2001) ACOG Practice Bulletin. Clinical management guidelines for obstetrician-gynecologists. Number 30, September 2001 (replaces Technical Bulletin Number 200, December 1994). Gestational diabetes. Obstet Gynecol, **98**:525-38.

10. Kim, C: (2010) Gestational diabetes: risks, management, and treatment options. Int J Womens Health, **2**:339-51.

11. Bellamy, L, Casas, JP, Hingorani, AD ,Williams, D: (2009) Type 2 diabetes mellitus after gestational diabetes: a systematic review and meta-analysis. Lancet, **373**:1773-9.

12. Tobias, DK, Zhang, C, van Dam, RM, Bowers, K ,Hu, FB: (2010) Physical activity before and during pregnancy and risk of gestational diabetes mellitus: a meta-analysis. Diabetes Care,

13. Stafne, SN, Salvesen, KA, Romundstad, PR, Eggebo, TM, Carlsen, SM ,Morkved, S: (2012) Regular exercise during pregnancy to prevent gestational diabetes: a randomized controlled trial. Obstet Gynecol, **119**:29-36.

14. (2003) Exercise during pregnancy and the postpartum period. Clin Obstet Gynecol, **46**:496-9.

15. Hinton, PS ,Olson, CM: (2001) Predictors of pregnancy-associated change in physical activity in a rural white population. Matern Child Health J, **5**:7-14.

16. Clapp, JF, 3rd ,Dickstein, S: (1984) Endurance exercise and pregnancy outcome. Med Sci Sports Exerc, **16**:556-62.

17. Andreasen, KR, Andersen, ML ,Schantz, AL: (2004) Obesity and pregnancy. Acta Obstet Gynecol Scand, **83**:1022-9.

18. Cnattingius, S, Bergstrom, R, Lipworth, L ,Kramer, MS: (1998) Prepregnancy weight and the risk of adverse pregnancy outcomes. N Engl J Med, **338**:147-52.

19. Clapp, JF, 3rd, Kim, H, Burciu, B, Schmidt, S, Petry, K ,Lopez, B: (2002) Continuing regular exercise during pregnancy: effect of exercise volume on fetoplacental growth. Am J Obstet Gynecol, **186**:142-7.

20. Hopkins, SA, Baldi, JC, Cutfield, WS, McCowan, L ,Hofman, PL: (2010) Exercise Training in Pregnancy Reduces Offspring Size without Changes in Maternal Insulin Sensitivity. J Clin Endocrinol Metab,

21. Clapp, JF, 3rd, Kim, H, Burciu, B ,Lopez, B: (2000) Beginning regular exercise in early pregnancy: effect on fetoplacental growth. Am J Obstet Gynecol, **183**:1484-8.

22. Garshasbi, A ,Faghih Zadeh, S: (2005) The effect of exercise on the intensity of low back pain in pregnant women. Int J Gynaecol Obstet, **88**:271-5.

23. Borg, G: (1970) Perceived exertion as an indicator of somatic stress. Scand J Rehabil Med, **2**:92-8.

24. Bahr, R, Aktivitetshåndboken: fysisk aktivitet i forebygging og behandling. [Oslo]: Helsedirektoratet; 2009.

25. Definition and diagnosis of diabetes mellitus and intermediate hyperglycemia. Geneve: International diabetes federation; 2006.

26. Christiansen, T, Paulsen, SK, Bruun, JM, Pedersen, SB ,Richelsen, B: (2010) Exercise training versus diet-induced weight-loss on metabolic risk factors and inflammatory markers in obese subjects: a 12-week randomized intervention study. Am J Physiol Endocrinol Metab, **298**:E824-31.

27. Wolff, S, Legarth, J, Vangsgaard, K, Toubro, S ,Astrup, A: (2008) A randomized trial of the effects of dietary counseling on gestational weight gain and glucose metabolism in obese pregnant women. Int J Obes (Lond), **32**:495-501.

28. Pinheiro, JC ,Bates, DM, Mixed-effects models in S and S-PLUS. New York: Springer; 2000.

29. Haskell, WL, Lee, IM, Pate, RR, Powell, KE, Blair, SN, Franklin, BA, Macera, CA, Heath, GW, Thompson, PD ,Bauman, A: (2007) Physical activity and public health: updated recommendation for adults from the American College of Sports Medicine and the American Heart Association. Circulation, **116**:1081-93.
