# Supplementary material for: Exercise Training and Weight Gain in Obese Pregnant Women: A Randomized Controlled Trial (ETIP Trial)
Source: PLoS Med. 2016 Jul 26;13(7):e1002079. doi: 10.1371/journal.pmed.1002079 (PMC4961392; doi:10.1371/journal.pmed.1002079)

**TRENING I SVANGERSKAPET**

**En randomisert klinisk studie av trening av gravide med bmi ≥ 28**

Du har blitt trukket ut til å delta i treningsgruppa i studien. Dette innebærer at du skal møte til trening ved St.Olavs hospital minst to ganger i uka. Treningen starter så fort som mulig etter de første testene. Treningen foregår ved Akutten-Hjerte-Lunge-senteret, 1.etasje, samme sted som en del av testingen ble gjort (grønt område i Sør-fløy, se kart neste side).

Tidspunkt for treningen avtales med den enkelte. Vi kan være behjelpelig med å snakke med arbeidsgiver om mulighet for å få trene i arbeidstiden.


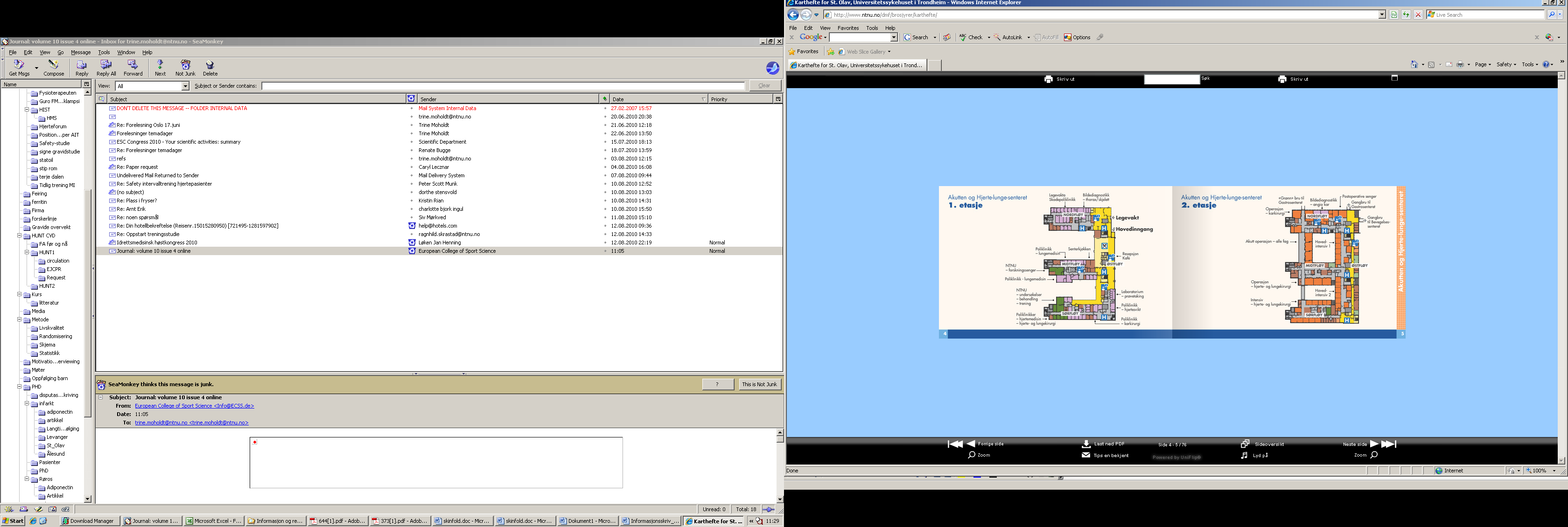

Supplement: S4 Text — (DOC) [file pmed.1002079.s009.doc]
