# Supplementary material for: Exercise Training and Weight Gain in Obese Pregnant Women: A Randomized Controlled Trial (ETIP Trial)
Source: PLoS Med. 2016 Jul 26;13(7):e1002079. doi: 10.1371/journal.pmed.1002079 (PMC4961392; doi:10.1371/journal.pmed.1002079)
Supplement: S5 Text — (DOC) [file pmed.1002079.s010.doc]

**Trening på sykehuset**

| Dato | Fart | Stigning | Distanse | Borg | Puls | Styrketrening, kommentarer | Trener |
| --- | --- | --- | --- | --- | --- | --- | --- |
|  |  |  |  |  |  |  |  |
|  |  |  |  |  |  |  |  |
|  |  |  |  |  |  |  |  |
|  |  |  |  |  |  |  |  |
|  |  |  |  |  |  |  |  |
|  |  |  |  |  |  |  |  |
|  |  |  |  |  |  |  |  |
|  |  |  |  |  |  |  |  |
|  |  |  |  |  |  |  |  |
|  |  |  |  |  |  |  |  |
|  |  |  |  |  |  |  |  |
|  |  |  |  |  |  |  |  |
|  |  |  |  |  |  |  |  |
|  |  |  |  |  |  |  |  |
|  |  |  |  |  |  |  |  |
