# Supplementary material for: Exercise Training and Weight Gain in Obese Pregnant Women: A Randomized Controlled Trial (ETIP Trial)
Source: PLoS Med. 2016 Jul 26;13(7):e1002079. doi: 10.1371/journal.pmed.1002079 (PMC4961392; doi:10.1371/journal.pmed.1002079)
Supplement: S6 Text — (DOC) [file pmed.1002079.s011.doc]

**TRENINGSPROGRAM**

**Dette programmet utføres på fellestreninger på St.Olav**

| Hvorfor | Tid | Hva | Beskrivelse |
| --- | --- | --- | --- |
| Oppvarming | 10 min | Gange på tredemølle | Du skal bli god og varm, og lett andpusten.  Borgs skala:12-13 |
| Utholdenhet | 25 min | Gange på tredemølle | Du skal kjenne at du trener, men klare å snakke litt underveis. Borgs skala: 13-15 |
| Bevegelighet | 2-3 min | Lette tøyninger | Tøy ut på baksida og forsida av lår, bakside legger. |
| Styrke bein | 2-3 min | Knebøy | Stå med hoftebreddes avstand mellom beina og bøy ned så langt du klarer i knærne.  10 repetisjoner x 3 |
| Bekkenbunn | 3 min | Stående løft | 10 repetisjoner (hold 6-8 sek + raske løft mot slutten av holdeperioden) |
| Styrke rygg + mage | 5 min | Diagonal-løft  +  Planken | Stå på alle fire og løft motsatt arm og ben.  10 repetisjoner hver side x 3  +  Knær eller tær og underarmer i gulvet. Rett linje fra skulder til kne eller ankel.  Hold i 30 sek. |
| Bekkenbunn | 3 min | Firefotstående | 10 repetisjoner (hold 6-8 sek + raske løft mot slutten av holdeperioden) |
| Styrke overkropp | 3 min | Armheving | Knær eller tær i gulvet. Strak rygg.  10 repetisjoner x 3 |
| Styrke mage | 3 min | Skrå sit-ups* | Ryggliggende: skulder mot motsatt kne.  10 repetisjoner på hver side, x 3. Trekk sammen bekkenbunnsmuskler før løft av overkroppen. |
| Bekkenbunn | 3 min | Sittende | 10 repetisjoner (hold 6-8 sek + raske løft mot slutten av holdeperioden) |

Totalt 60 min

******* Kan være ubehagelig for noen pga avklemming av vena cava – da utføres øvelsen i sittende (skredderstilling, motstand mot rotasjon annenhver side). Hvis ubehaglig nedtrykksfølelse i bekkenet / underlivet – IKKE gjør øvelsen.
