# Supplementary material for: Exercise Training and Weight Gain in Obese Pregnant Women: A Randomized Controlled Trial (ETIP Trial)
Source: PLoS Med. 2016 Jul 26;13(7):e1002079. doi: 10.1371/journal.pmed.1002079 (PMC4961392; doi:10.1371/journal.pmed.1002079)
Supplement: S8 Text — (DOC) [file pmed.1002079.s013.doc]

**EGENTRENING**

Egentreningsprogrammet består av to deler, både utholdenhetstrening (30 min) og styrketrening (ca 15 min).

Utholdenhetstreningen kan oppnås på ulike måter, men det er viktig at du finner en treningsform som passer deg. Du kan gå rask tur, jogge, sykle, svømme eller annet. Det viktigste er at du får økt puls over lengre tid (30 min). Intensiteten skal være såpass at det oppleves ***litt anstrengende***, det vil si at du skal bli varm og svett.

Styrketreningen har til hensikt å styrke ben, armer, rygg, mage og bekkenbunnsmuskler. Det er viktig at du prøver deg litt fram og finner den utgangsstillingen som passer best for deg. Spør gjerne personen som leder gruppetreningen om hjelp til dette. Det vil være naturlig at en utgangsstilling som passer deg bra i begynnelsen av treningsperioden, passer mindre bra mot slutten av treningsperioden enten fordi magen har blitt større, du har blitt sterkere eller noe oppleves ubehagelig. Da kan du bytte til en annen utgangsstilling.

Du skal ta ***10 repetisjoner x 3***

Underveis eller etter treningen skal du ikke ha noe ubehag. Det er naturlig å være litt støl i etterkant av treningen, spesielt i oppstartsfasen, men du skal ikke ha smerter. Ta kontakt dersom noe medfører ubehag.

Hjemmetreningsprogrammet skal gjøres minst ***to dager i uka*** i tillegg til treninga på sykehuset. Det er fint om du er så aktiv som mulig i hverdagen utover denne treninga også.

OBS! Husk å registrere treninga i treningsdagboka!

**Styrketrening**

KNEBØY


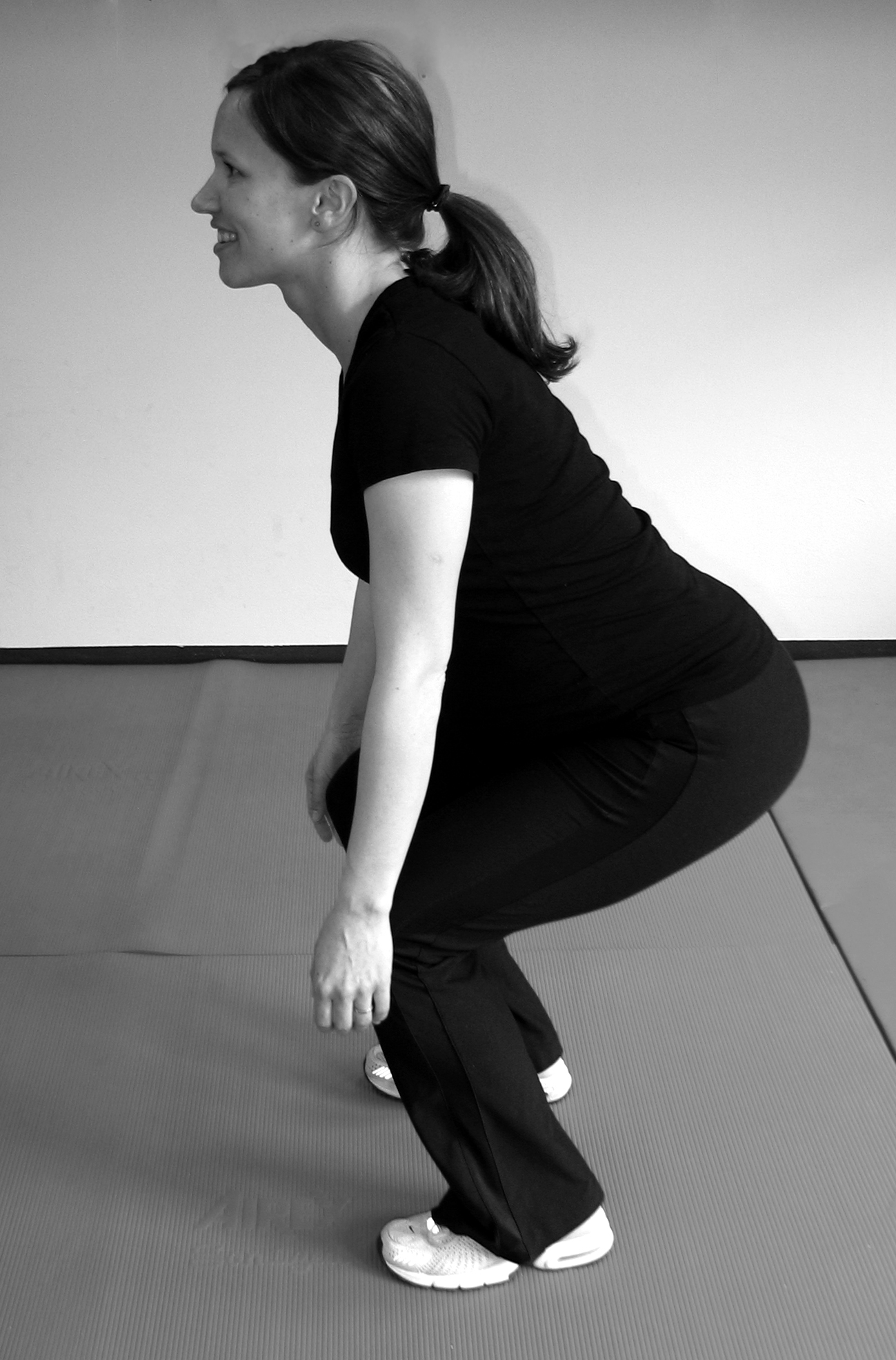


Stå med hoftebreddes avstand med føttene. Bøy ned i hofte- og kneledd med ”knær over tær”, og rumpa ut bak som om du skal sette deg på en stol. Stram setet og strekk opp igjen til stående stilling. Varier tyngden med hvor langt du bøyer ned.

10 repetisjoner x 3

”PLANKEN” (gjøres tre ganger)


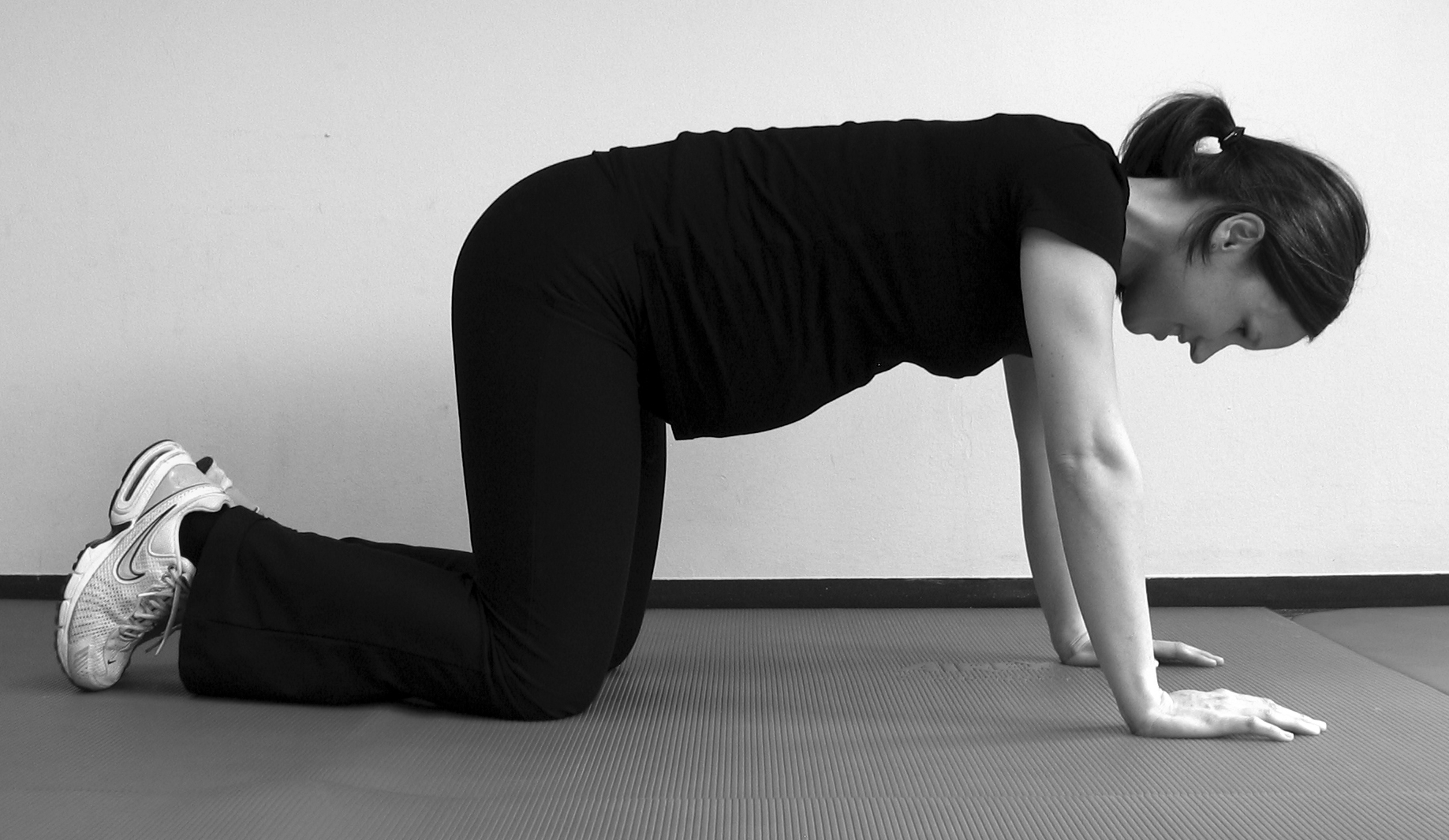


Alternativ A)

Stå på alle fire (knær og strake armer) med ryggen i nøytral stilling. Trekk navlen inn mot ryggen (nedre del av magen) og hold posisjonen i 30 sek.


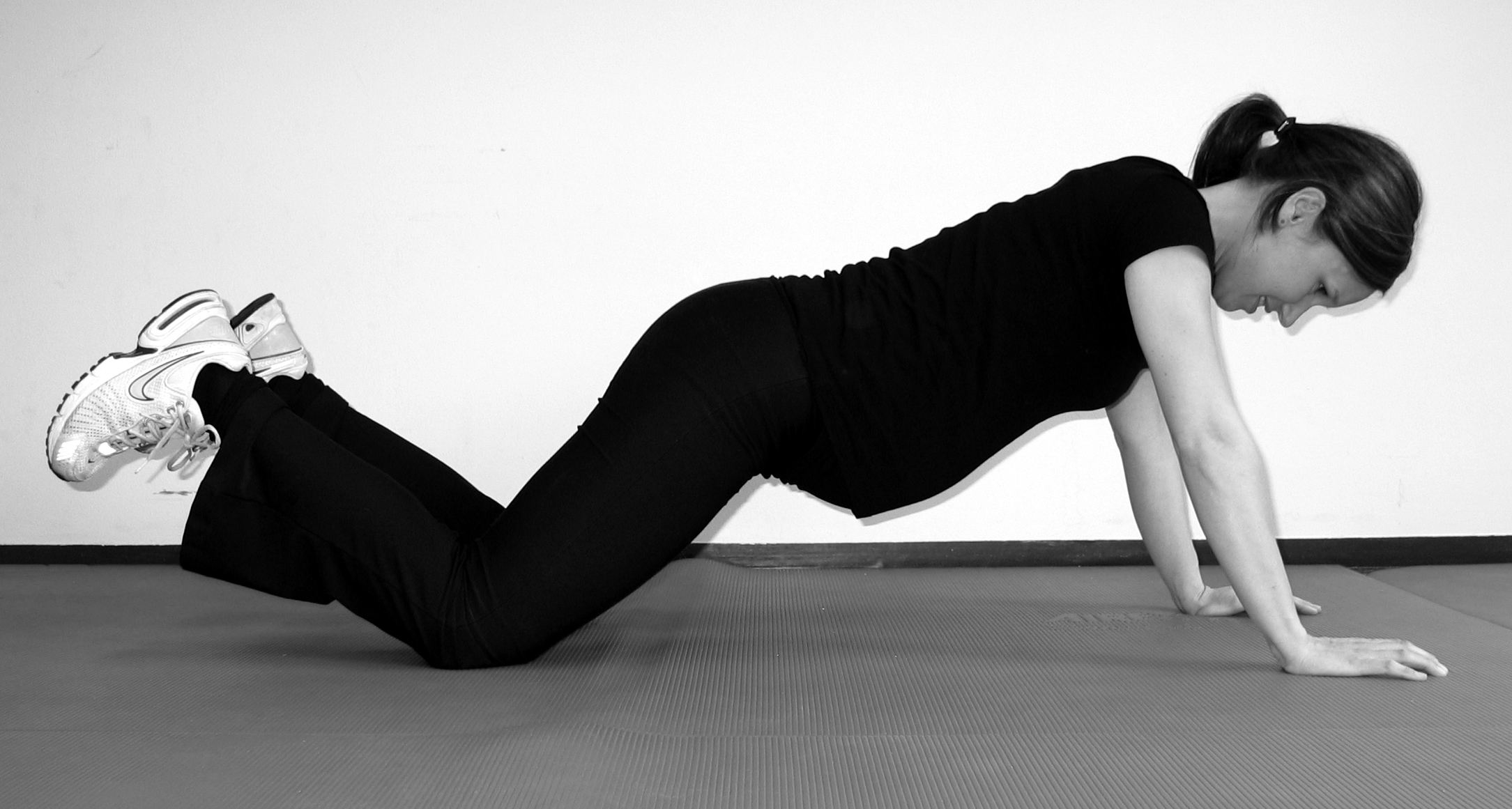


Alternativ B)

Stå på knær og strake armer, eller ha underarmer i gulvet. Hold kroppen strak og ryggen i nøytral stilling. Trekk navlen inn mot ryggen (nedre del av magen) og hold posisjonen i 30 sek.


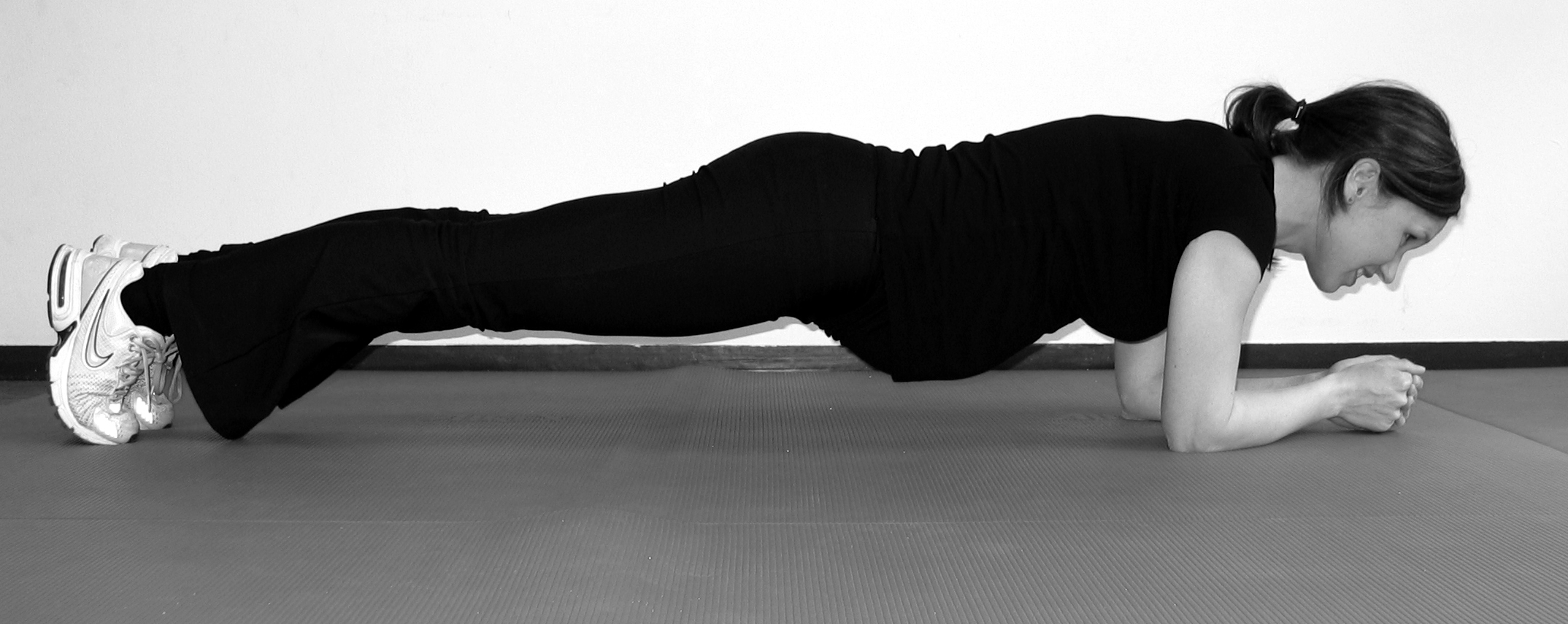
Alternativ C)

Stå på tærne og albuene, hold kroppen strak og ryggen i nøytral stilling.

Trekk navlen inn mot ryggen (nedre del av magen) og finn stillingen som bildet viser:

Hold posisjonen i 30 sek.

**OBS!**

**Pust godt mens du gjør øvelsen (ikke hold pusten)!**

**Dersom du ikke klarer å holde posisjonen må du gå bytte til en enklere utgangsstilling eller korte ned holdetiden!**

ARMHEVINGER


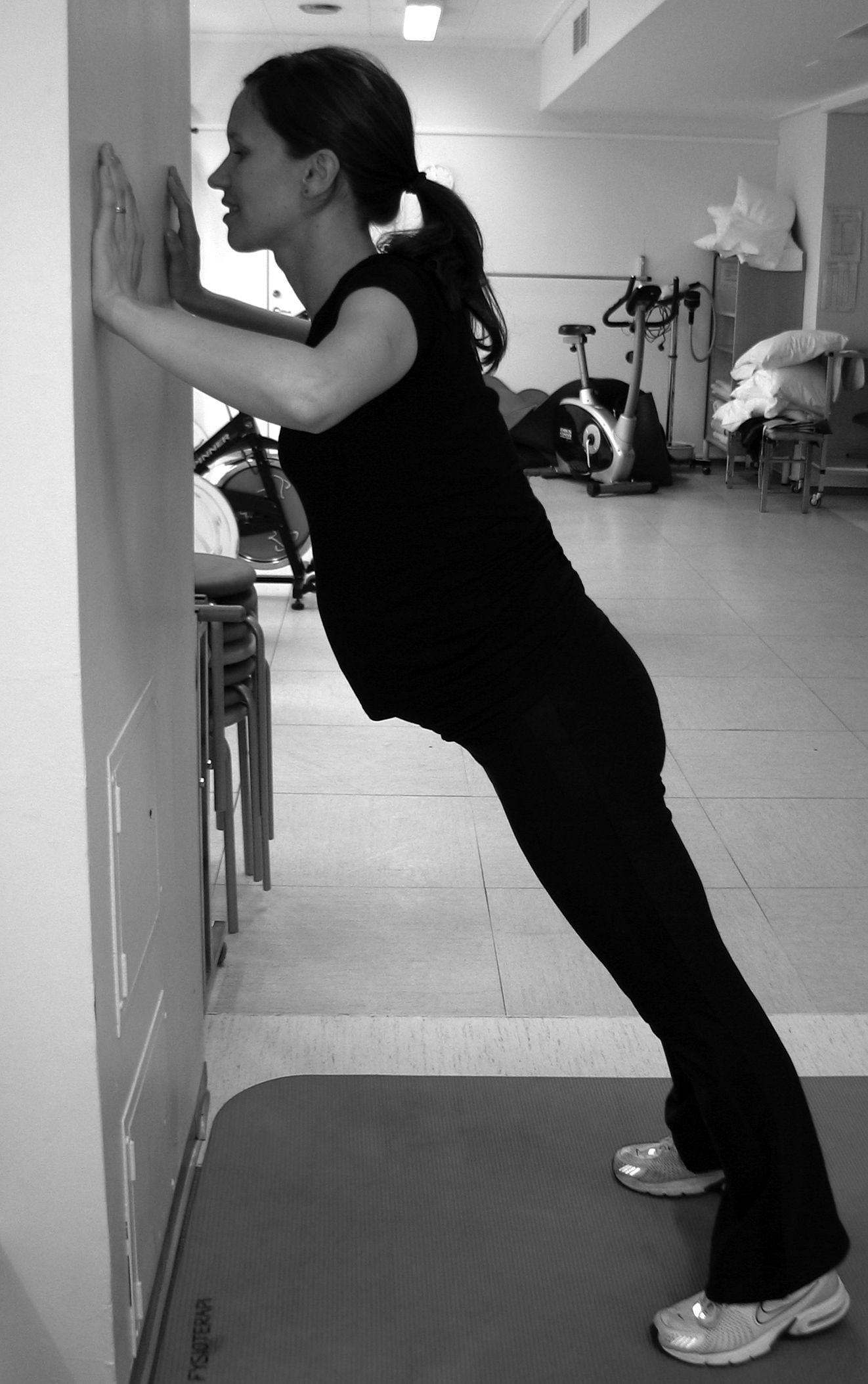


Alternativ A)

Stå med føttene i skulderbreddes avstand et stykke ut fra veggen. Len deg mot veggen på strake armer med strak kropp. Trekk navlen inn mot ryggen (nedre del av magen, se ”Plankeøvelsen”). Gjør armhevinger mot veggen. Varier tyngden med å stå nærmere veggen (lettere) eller lenger fra veggen (tyngre).

10 repetisjoner x 3


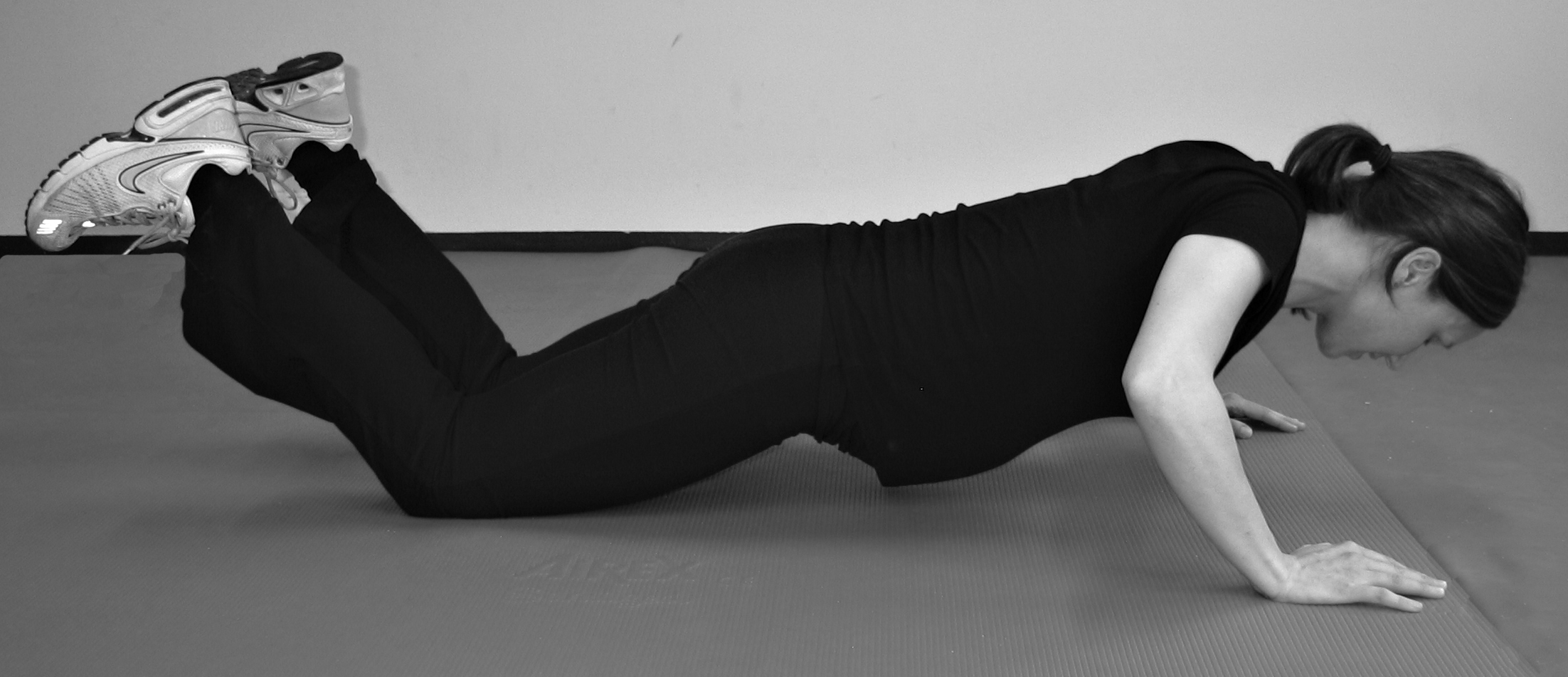


Alternativ B)

Stå på knær og strake armer. Trekk navlen inn mot ryggen (nedre del av magen, se ”Plankeøvelsen”).

Gjør armhevinger med knærne i gulvet og strak kropp.


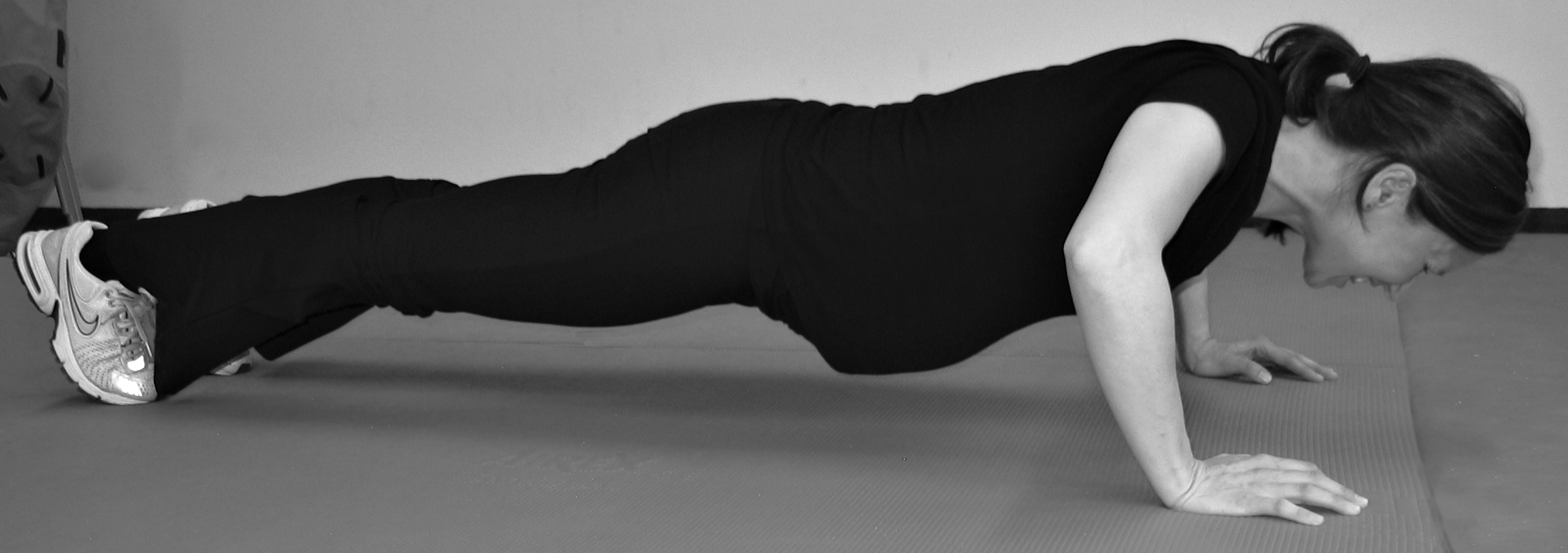
Alternativ C)

Stå på tær og strake armer. Trekk navlen inn mot ryggen (nedre del av magen, se ”Plankeøvelsen”). Gjør armhevinger med tærne i gulvet og strak kropp.

DIAGONALLØFT


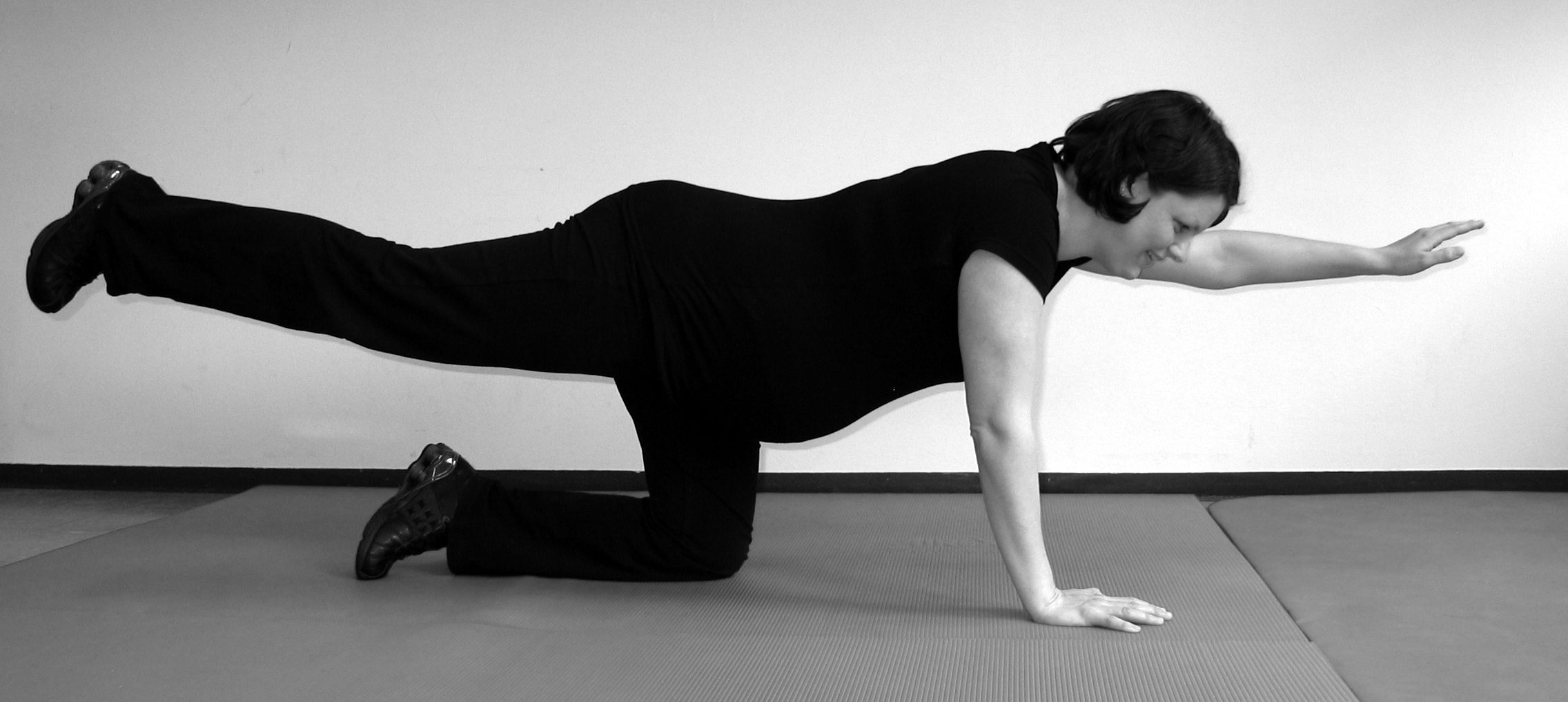


Alternativ A)

Stå på knær og strake armer med hodet i nøytral stilling. Trekk navlen inn mot ryggen (nedre del av magen, se ”Plankeøvelsen”). Løft diagonalt arm og ben til vannrett stilling.

10 repetisjoner x 3

Alternativ ved smerter:


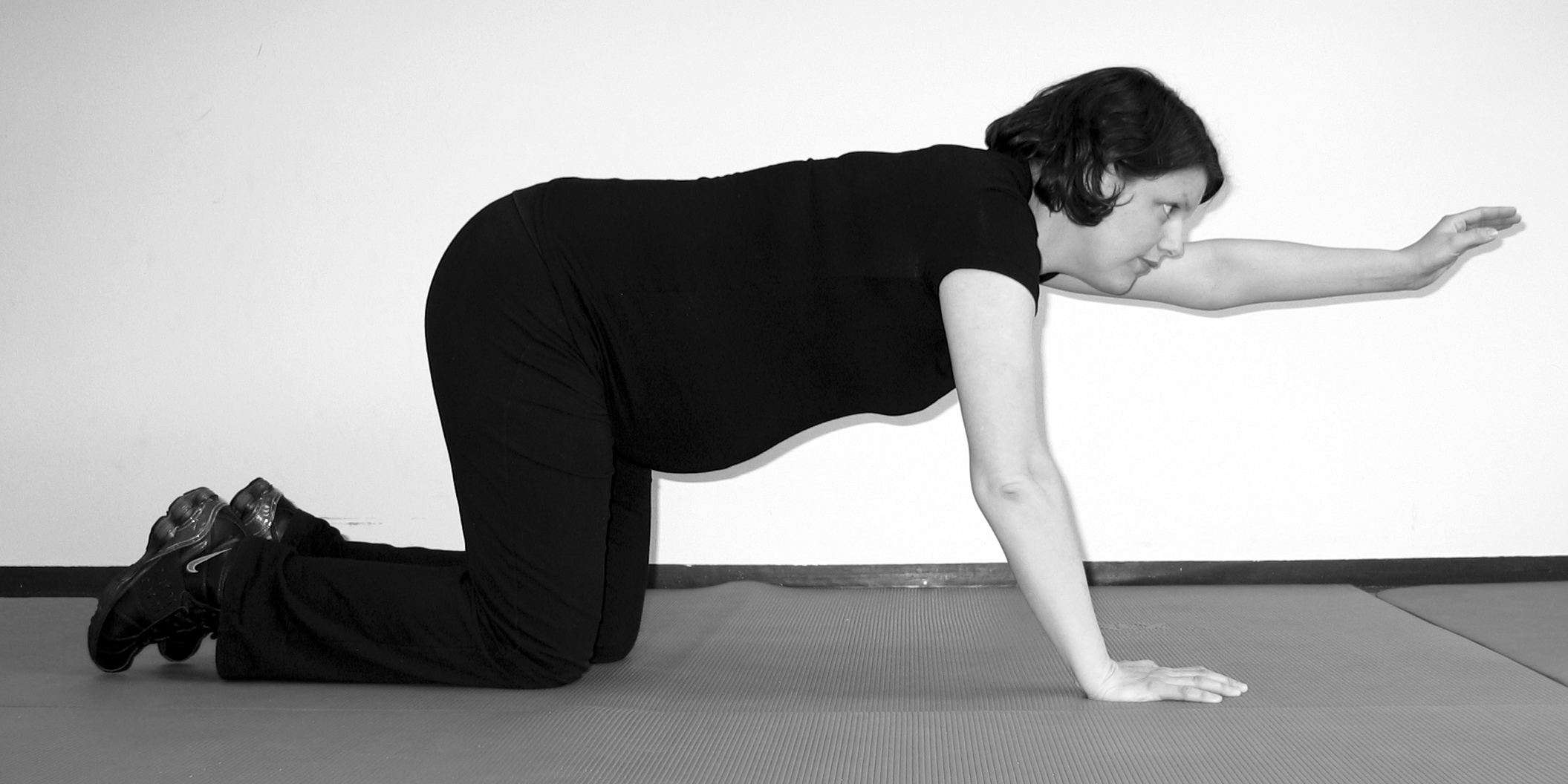


Alternativ B)

Stå på knær og strake armer med hodet i nøytral stilling. Trekk navlen inn mot ryggen (nedre del av magen, se ”Plankeøvelsen”). Løft en og en arm strakt frem.


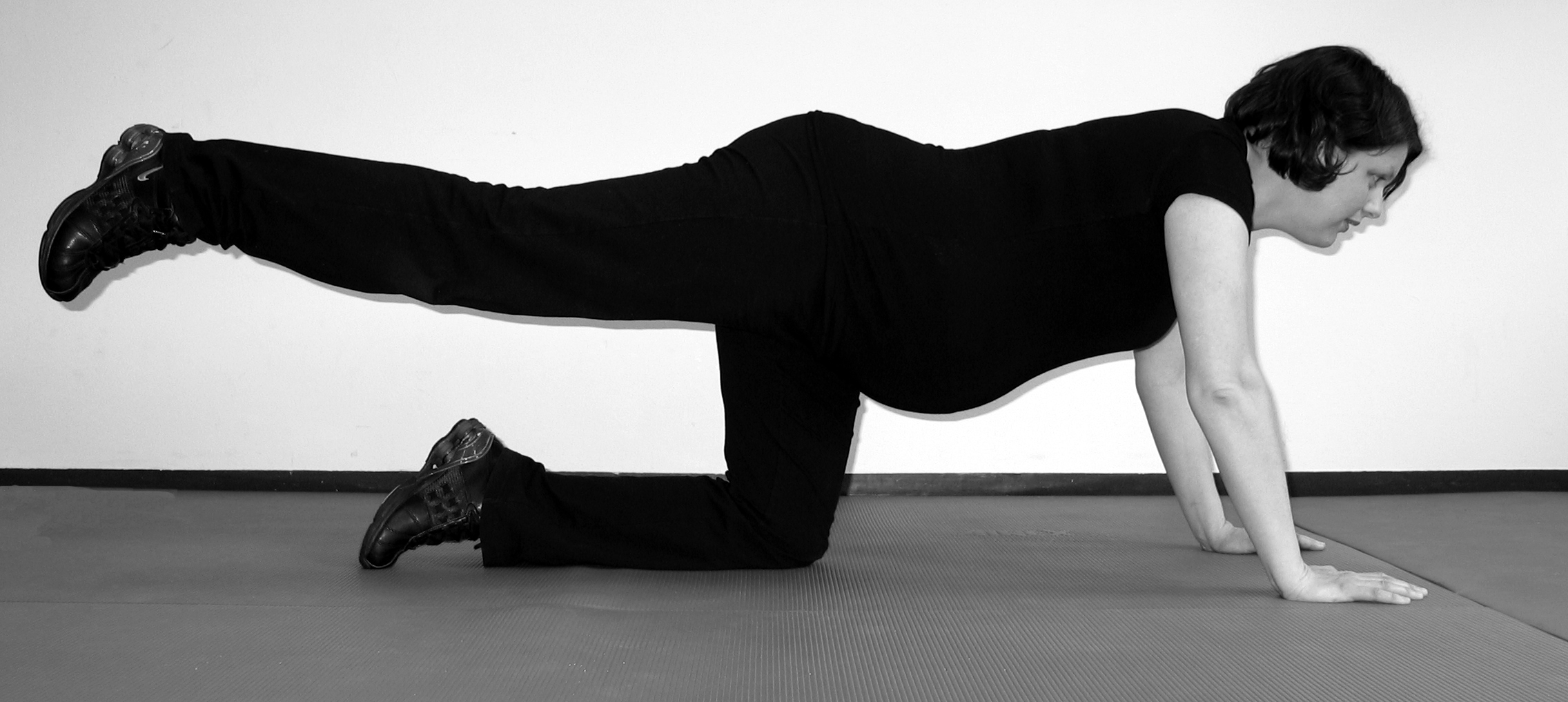

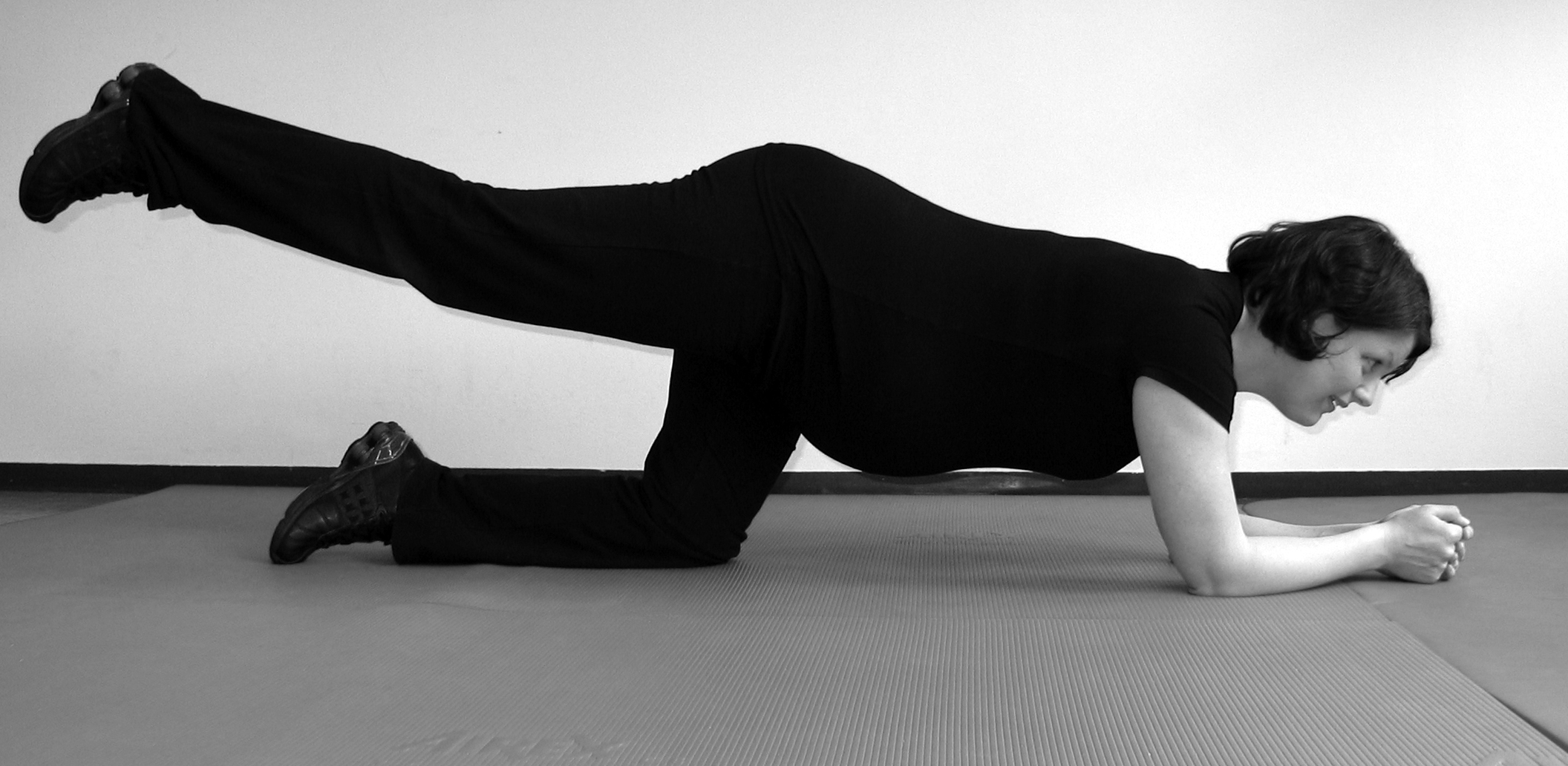


Alternativ C)

Stå på knær og strake armer eller på albuene med hodet i nøytral stilling. Trekk navlen inn mot ryggen (nedre del av magen, se ”Plankeøvelsen”). Strekk et og et ben vannrett bakover.

BEKKENBUNNENS MUSKLER

Løft opp og inn rundt urinrør, skjede og endetarm uten å spenne mage, sete og lår. Ta i så hardt du kan under hver sammentrekning og forsøk å holde i 6-8 sekunder før du slipper rolig ned. Pust rolig ut og inn, både under og mellom muskelsammentrekningene. Velg en eller flere av disse utgangsstilingene:


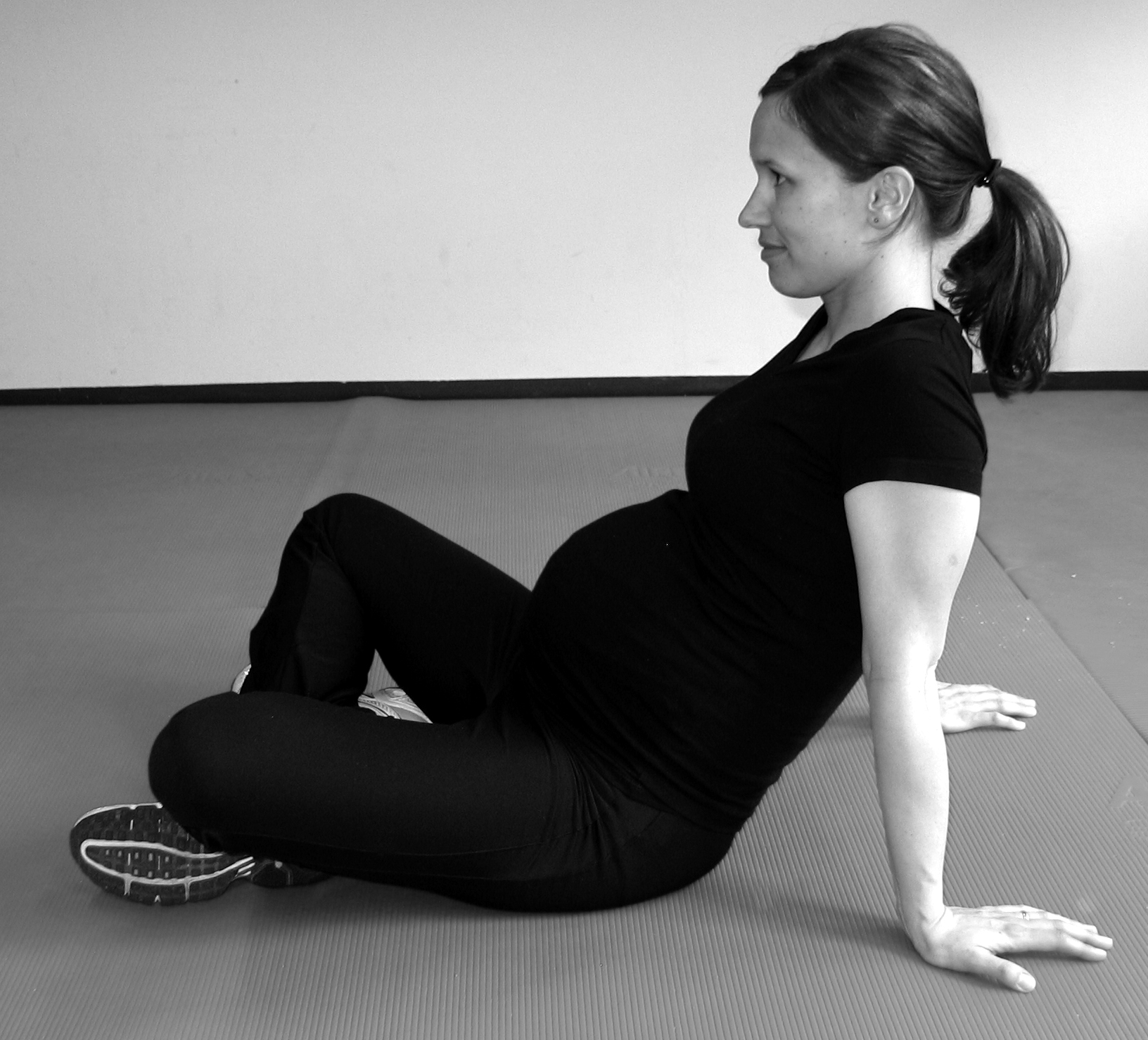


Alternativ A)

Sitt med bena fra hverandre i skredderstilling med rett rygg. Trekk sammen rundt åpningene i bekkenbunnen.


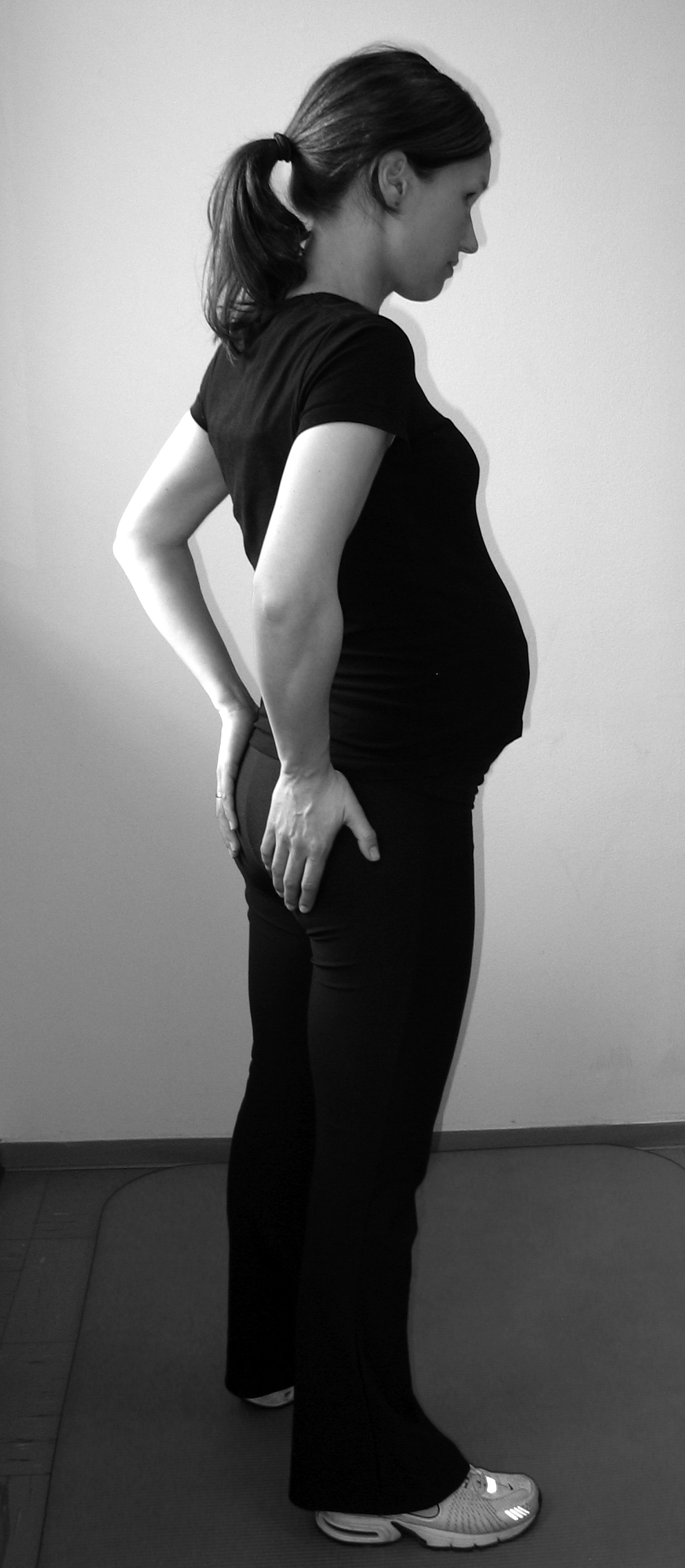


Alternativ B)

Stå med bena fra hverandre, kjenn etter at du er slapp i setemusklene mens du trekker sammen i bekkenbunnsmusklene.


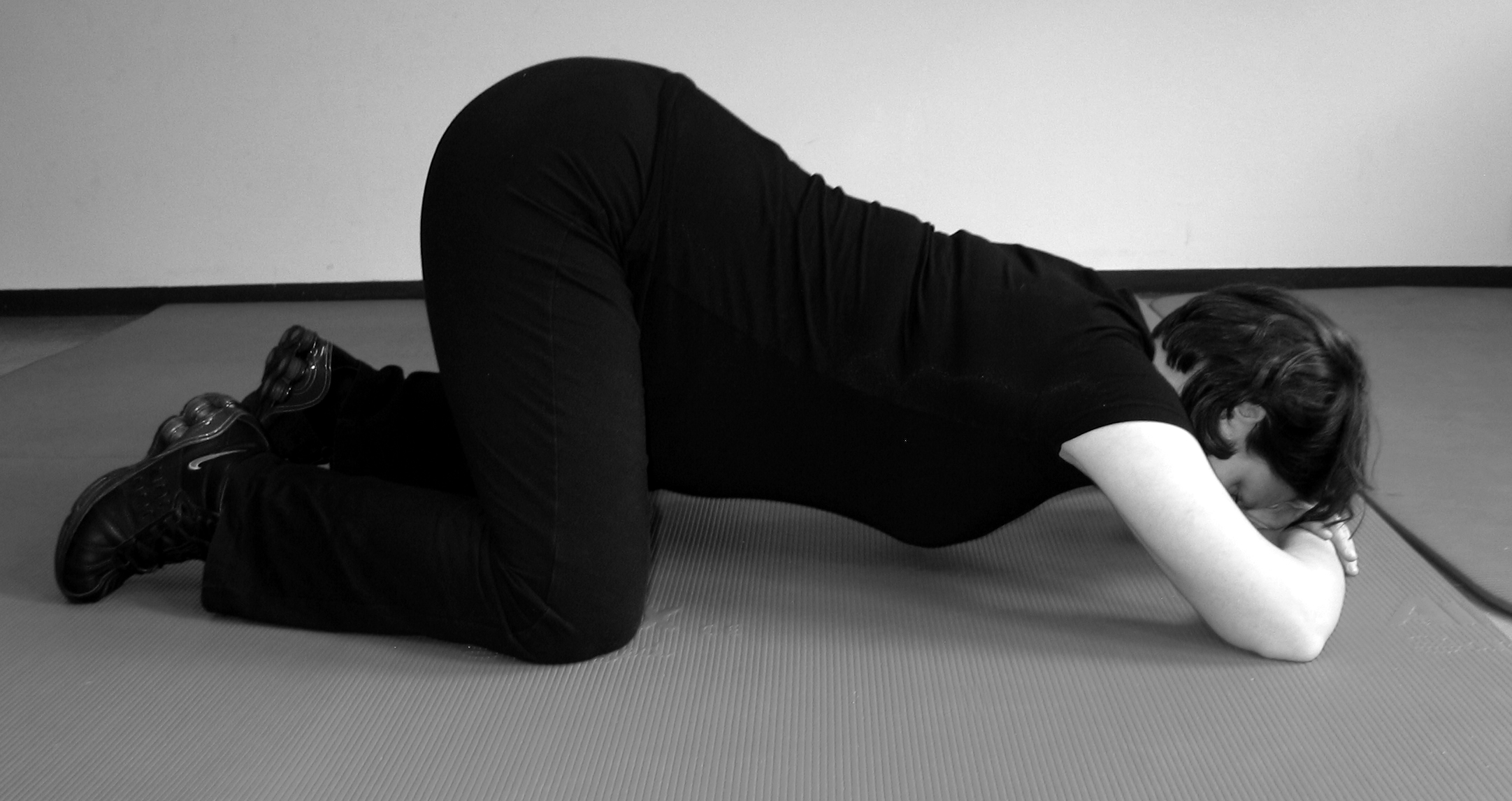
Alternativ C)

Stå på alle fire med knærne ut til siden og føttene sammen. Hev bekkenbunnen opp og innover.

SKRÅ MAGEMUSKLER


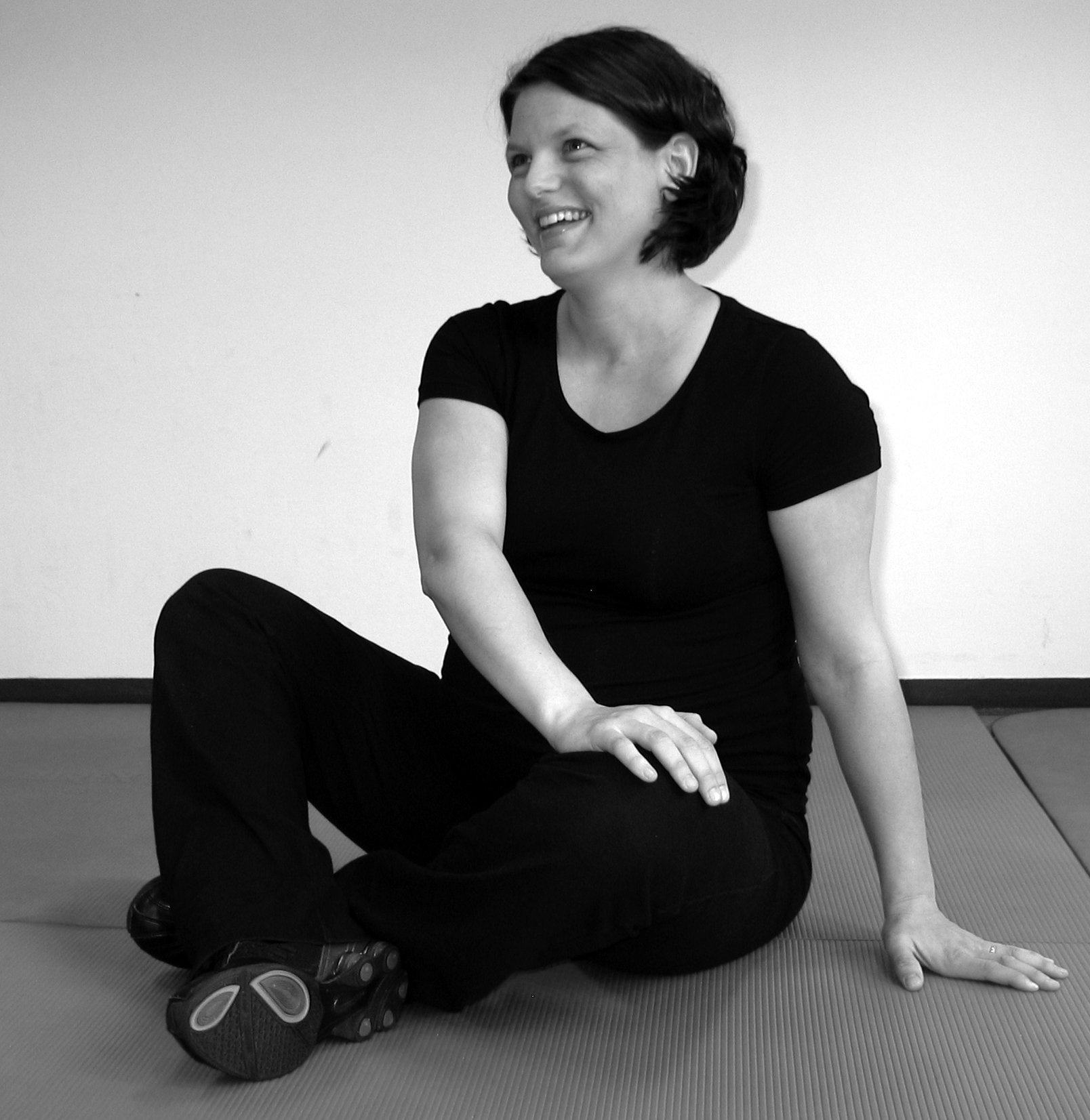
Alternativ A)

Skrå sit-ups i sittende. Sitt i skredderstilling med rett rygg. Press motsatt arm og kne mot hverandre. Bruk strak arm og press diagonalt.

10 repetisjoner til hver side, x 3


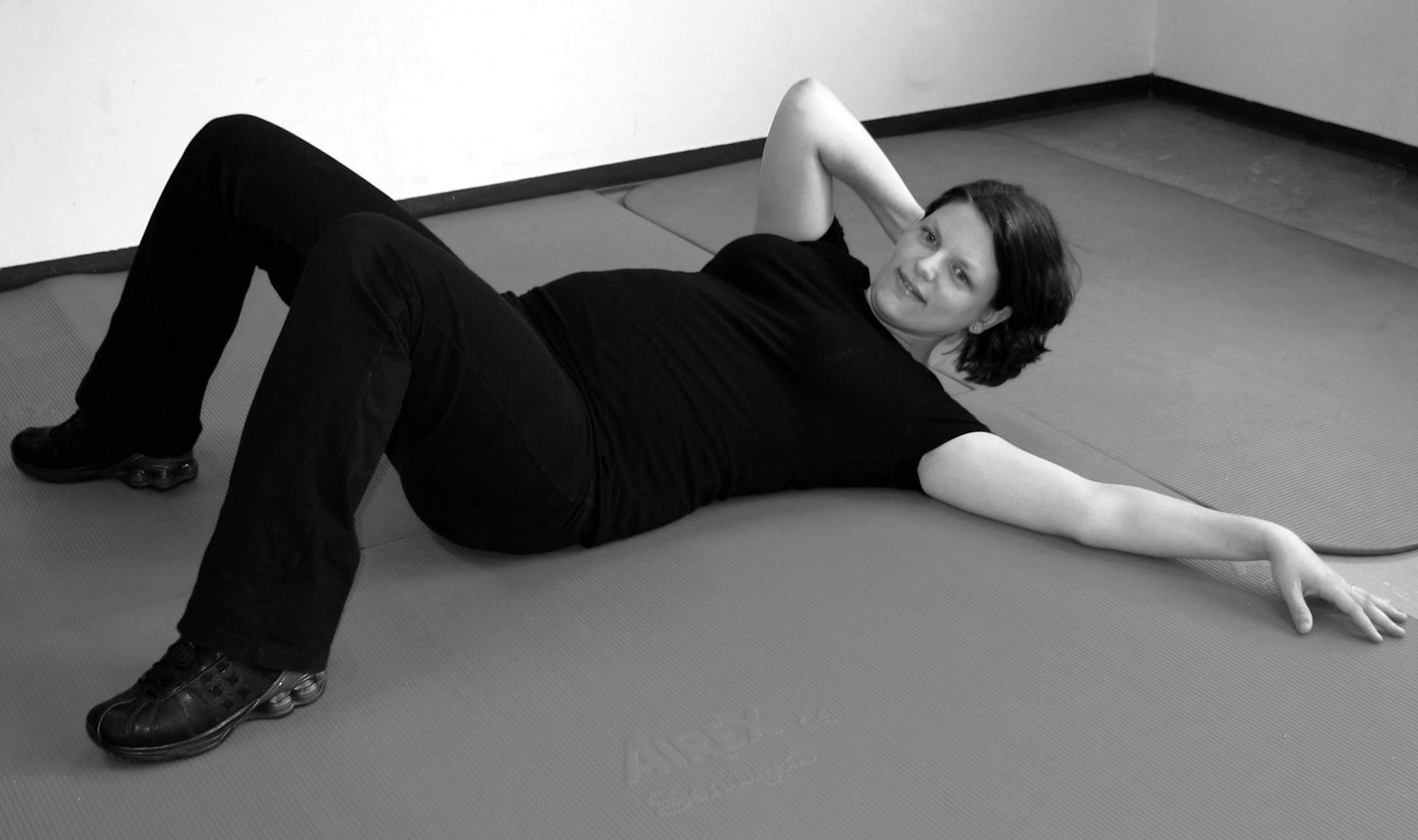

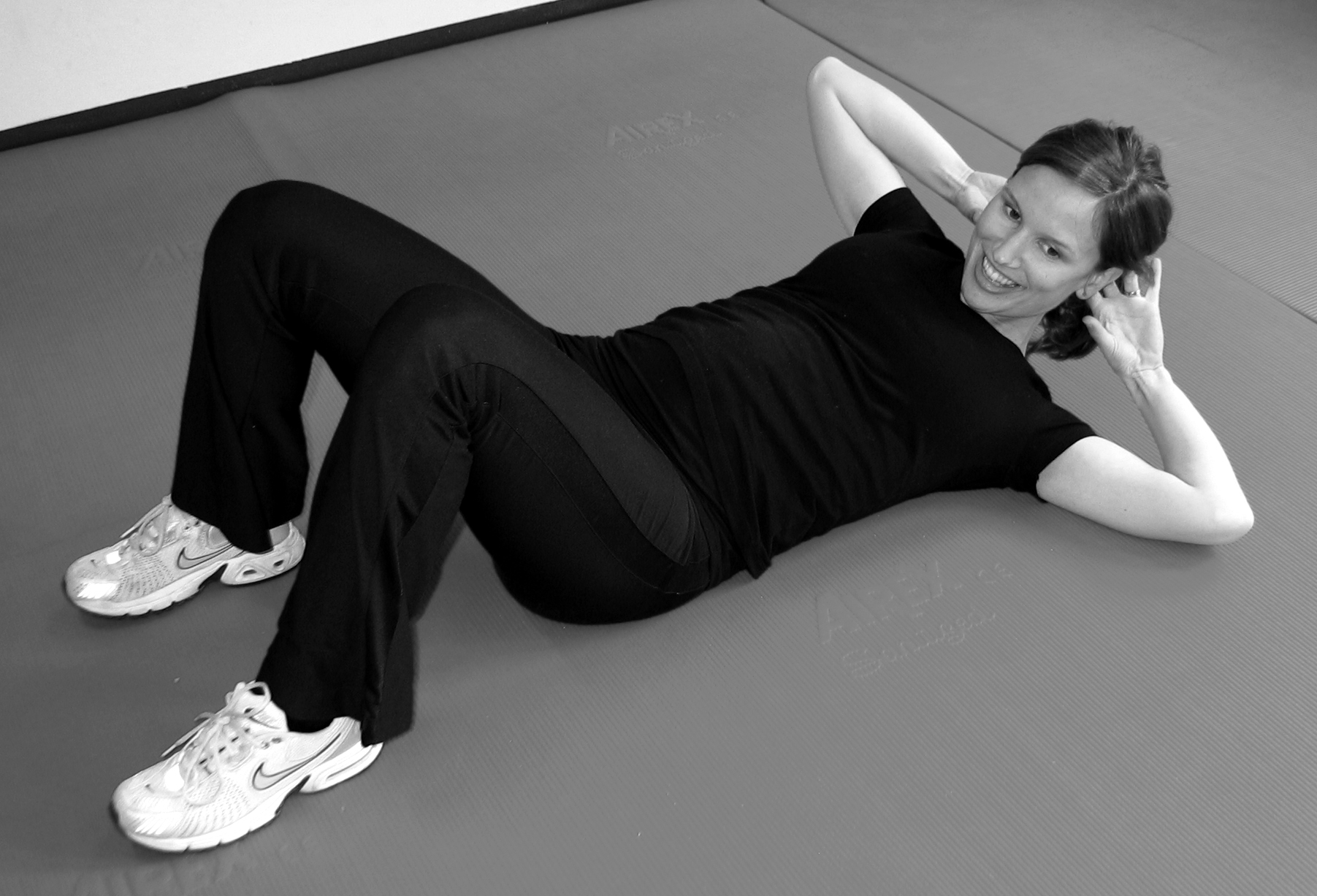


Alt. b)

Skrå sit-ups i ryggliggende. Ligg på ryggen med bøyde ben og korsryggen i kontakt med gulvet. Løft overkroppen litt opp fra underlaget slik at skulder peker mot motsatt kne.

**OBS!**

**Noen gravide kan bli uvel eller svimmel av å ligge på ryggen. Dersom det gjelder deg bør du trene de skrå bukmusklene i utgangsstilling a.**

Trekk sammen bekkenbunnsmusklene samtidig som du gjør skrå sit-ups.
