# Supplementary material for: Exercise Training and Weight Gain in Obese Pregnant Women: A Randomized Controlled Trial (ETIP Trial)
Source: PLoS Med. 2016 Jul 26;13(7):e1002079. doi: 10.1371/journal.pmed.1002079 (PMC4961392; doi:10.1371/journal.pmed.1002079)
Supplement: S9 Text — (PDF) [file pmed.1002079.s014.pdf]

FYSISK AKTIVITET OG TRENING

For å få en oversikt over ditt aktivitetsnivå før dette aktuelle svangerskapet ber vi om at du besvarer spørsmålene nedenfor som handler om fysisk aktivitet og trening.

Mosjon og fysisk aktivitet på fritiden.
Fysisk aktivitet på fritiden kan deles inn i 3 hovedtyper; 1) gåturer, 2) moderat anstrengende aktiviteter og 3) meget anstrengende aktiviteter. Hvis eksemplene ikke dekker dine aktiviteter; kryss i den kategorien som passer best med hvor anstrengende aktiviteten har vært.

1) TURGÅING

a) Hvor mange dager i uken gikk du turer siste året før du ble gravid?

☐ 0 ☐ 1 ☐ 2 ☐ 3 ☐ 4 ☐ 5 ☐ 6 ☐ 7

b) Hvor lenge gikk du per gang (i gjennomsnitt)?

☐ Under 5 min	☐ 20-29 min	☐ 1-1,5 t
☐ 5-9 min	☐ 30-44 min	☐ 1,5-2 t
☐ 10-19 min	☐ 45-59 min	☐ Over 2 t

c) Når du gikk tur på fritiden; i hvilket tempo gikk du (i gjennomsnitt)?

☐ Sakte tempo ☐ Moderat tempo ☐ Raskt tempo

2) MIDDELS ANSTRENGENDE AKTIVITETER

a) Hvor mange dager i uken siste året før du ble gravid utførte du middels anstrengende aktiviteter som for eksempel å sykle i moderat tempo, svømme i moderat tempo, jogge rolig, rolige skiturer, dans, golf etc. på fritiden?

☐ 0 ☐ 1 ☐ 2 ☐ 3 ☐ 4 ☐ 5 ☐ 6 ☐ 7

b) Hvor lenge varte aktiviteten per gang (i gjennomsnitt)?

☐ Under 5 min	☐ 20-29 min	☐ 1-1,5 t
☐ 5-9 min	☐ 30-44 min	☐ 1,5-2 t
☐ 10-19 min	☐ 45-59 min	☐ Over 2 t

3) MEGET ANSTRENGENDE AKTIVITETER

a) Hvor mange dager i uken siste året før du ble gravid utførte du meget anstrengende aktiviteter som for eksempel aerobics, løping, sykle fort, svømme fort, gå raskt på ski, ballspill etc. på fritiden?

☐ 0 ☐ 1 ☐ 2 ☐ 3 ☐ 4 ☐ 5 ☐ 6 ☐ 7

b) Hvor lenge varte aktiviteten per gang (i gjennomsnitt)?

☐ Under 5 min	☐ 20-29 min	☐ 1-1,5 t
☐ 5-9 min	☐ 30-44 min	☐ 1,5-2 t
☐ 10-19 min	☐ 45-59 min	☐ Over 2 t

4) TID DU TILBRINGER I RO

Hvor mye tid brukte du av fritiden på TV/PC/Internett/lese/slappe av per dag før du ble gravid (i gjennomsnitt)?

☐ 0 min	☐ 1-2 t	☐ 4-5 t
☐ 1-29 min	☐ 2-3 t	☐ Mer enn 5 timer
☐ 30-59 min	☐ 3-4 t	

ID-nummer: | | | |

DIN HELSE OG TRIVSEL

5. Hvordan vil du, stort sett, vurdere din helsetilstand i løpet av de siste 4 ukene?

☐ Utmerket ☐ Meget god ☐ God ☐ Nokså god ☐ Dårlig ☐ Svært dårlig

6. I løpet av de siste 4 ukene, i hvilken grad begrenset fysiske helseproblemer dine vanlige fysiske aktiviteter (spasere, gå opp trapper)?

☐ Ikke i det hele tatt ☐ Svært lite ☐ En del ☐ Mye ☐ Kunne ikke utføre fysiske aktiviteter

7. I løpet av de siste 4 ukene, hvor vanskelig var det for deg å utføre ditt vanlige arbeid (både i og utenfor hjemmet) på grunn av din fysiske helse?

☐ Ikke i det hele tatt ☐ Litt ☐ Nokså ☐ Meget ☐ Kunne ikke utføre daglig arbeid

8. Hvor sterke kroppslige smerter har du hatt i løpet av de siste 4 ukene?

☐ Ingen ☐ Meget svake ☐ Svake ☐ Moderate ☐ Sterke ☐ Meget sterke

9. I løpet av de siste 4 ukene, hvor mye overskudd hadde du?

☐ Svært mye ☐ Ganske mye ☐ En del ☐ Litt ☐ Ikke noe

10. I løpet av de siste 4 ukene, i hvilken grad begrenset din fysiske helse eller følelsesmessige problemer din vanlige sosiale omgang med familie eller venner?

☐ Ikke i det hele tatt ☐ Svært lite ☐ En del ☐ Mye ☐ Kunne ikke ha sosial omgang

11. I løpet av de siste 4 ukene, i hvilken grad har du vært plaget av følelsesmessige problemer (som f.eks. å være engstelig, deprimert eller irritabel)?

☐ Ikke i det hele tatt ☐ Litt ☐ En del ☐ Mye ☐ Svært mye

12. I løpet av de siste 4 ukene, i hvilken grad hindret personlige eller følelsesmessige problemer deg fra å utføre ditt vanlige arbeid, skolegang eller andre daglige gjøremål?

☐ Ikke i det hele tatt ☐ Svært lite ☐ En del ☐ Mye ☐ Kunne ikke utføre daglige gjøremål

SYKDOMMER I FAMILIEN

13) Har noen i din familie hatt svangerskapsforgiftning?

☐ Ja

☐ Nei *(gå til spørsmål 16)*

14) Hvem i din familie hadde svangerskapsforgiftning?

____

15) Hvor alvorlig var svangerskapsforgiftningen?

☐ Lett

☐ Alvorlig *(med for tidlig fødsel, redusert fostervekst)*

16) Har noen i din familie diabetes?

☐ Ja

☐ Nei *(gå til spørsmål 19)*

17) Hvem i din familie har diabetes?

____

18) Er dette diabetes type 1 eller diabetes type 2?

☐ Type 1

☐ Type 2

19) Har noen i din familie forhøyet blodtrykk eller hjertekar-sykdom?

☐ Ja

☐ Nei *((da trenger du ikke svare på flere spørsmål)*

20) Hvem i din familie har forhøyet blodtrykk eller hjertekar-sykdom?

____

21) Vet du ca alder når symptomene på forhøyet blodtrykk eller hjertekar-sykdom debuterte?

____
