# Supplementary material for: Exercise Training and Weight Gain in Obese Pregnant Women: A Randomized Controlled Trial (ETIP Trial)
Source: PLoS Med. 2016 Jul 26;13(7):e1002079. doi: 10.1371/journal.pmed.1002079 (PMC4961392; doi:10.1371/journal.pmed.1002079)
Supplement: S10 Text — (PDF) [file pmed.1002079.s015.pdf]

BAKGRUNNSINFORMASJON

1) Hvilken svangerskapsuke er du i NÅ? ☐☐

2) Hvordan vil du i alminnelighet vurdere din helse nå?

☐ Meget god
☐ God
☐ Verken god eller dårlig
☐ Nokså dårlig
☐ Dårlig

3) Røyker du?

☐ Ja, ca ☐☐ sigaretter daglig
☐ Ja, ca ☐☐ priser snus daglig
☐ Nei

4) Synes du din samboer/ektefelle tar sin del av arbeidsoppgavene i hjemmet?

☐ Helt enig ☐ Nokså enig ☐ Nokså uenig ☐ Helt uenig

5) Er det endringer i din arbeidsstatus siden sist? (Ikke ta hensyn til eventuell sykemelding)

☐ Ja ☐ Nei (gå videre til spørsmål 7)

6) Har du inntektsgivende arbeid?
(Regn også med midlertidig ansettelse og kortvarende arbeid. Ikke ta hensyn til om du er sykmeldt eller har permisjon, bare om du har arbeid. Som inntektsgivende arbeid regnes også arbeid uten fast lønn i bedrift som familien eier, f.eks. gårdsbruk, forretning)

☐ Ja, hvor stor stillingsandel er du ansatt i? ☐☐☐ %

☐ Nei, jeg er ☐ Student/skoleelev
☐ Arbeidsufør
☐ Hjemmeværende
☐ Arbeidsledig

7) Har du fått hjelp av din arbeidsgiver til å tilrettelegge arbeidsdagen din?

☐ Ja, jeg har fått ☐ Endrede arbeidsoppgaver
☐ Flere pauser
☐ Endring av vakter
☐ Hjelpemidler
☐ Annet:________________________

☐ Nei, det var ikke mulig å få til endringer i min arbeidsdag
☐ Nei, det behøves ikke

8) Synes du hjelpen du har fått av arbeidsgiver med tilrettelegging har vært tilstrekkelig?

☐ Ja ☐ Nei

9) Har du vært sykmeldt siden du var her på forrige test?

☐ Ja ☐ Nei (gå videre til spørsmål 11)

☐ Ja, jeg var sykmeldt ved første test og er fortsatt sykmeldt i ☐☐☐ **%**

10) Dersom du har vært sykmeldt under dette svangerskapet, ber vi deg spesifisere nedenfor om din/dine sykmeldinger:

a) Første sykmeldingsperiode:

Prosent sykmeldt: ☐☐☐ %

fra (ddmmåååå): ☐☐.☐☐.☐☐☐☐ til (ddmmåååå): ☐☐.☐☐.☐☐☐☐

Var du i denne perioden aktivt sykmeldt? ☐ Ja ☐ Nei

Årsak til sykmelding:

☐ Bekkensmerter / bekkenløsning
☐ Ryggsmerter
☐ Falske rier / kynnere
☐ Kvalme / oppkast
☐ Søvnvansker
☐ Slitenhet / tretthet
☐ Blodtrykk
☐ Annet: __________________

b) Andre sykmeldingsperiode:

Prosent sykmeldt: ☐☐☐ %

fra (ddmmåååå): ☐☐.☐☐.☐☐☐☐ til (ddmmåååå): ☐☐.☐☐.☐☐☐☐

Var du i denne perioden aktivt sykmeldt? ☐ Ja ☐ Nei

Årsak til sykmelding:

☐ Bekkensmerter / bekkenløsning
☐ Ryggsmerter
☐ Falske rier / kynnere
☐ Kvalme / oppkast
☐ Søvnvansker
☐ Slitenhet / tretthet
☐ Blodtrykk
☐ Annet: __________________

c) Tredje sykmeldingsperiode:

Prosent sykmeldt: ☐☐☐ %

fra (ddmmåååå): ☐☐.☐☐.☐☐☐☐ til (ddmmåååå): ☐☐.☐☐.☐☐☐☐

Var du i denne perioden aktivt sykmeldt? ☐ Ja ☐ Nei

Årsak til sykmelding:

☐ Bekkensmerter / bekkenløsning
☐ Ryggsmerter
☐ Falske rier / kynnere
☐ Kvalme / oppkast
☐ Søvnvansker
☐ Slitenhet / tretthet
☐ Blodtrykk
☐ Annet: __________________

d) Eventuelle kommentarer:

____

____

____

e) Antall egenmeldingsdager i løpet av svangerskapet (dager i tillegg til sykemeldingsperioder): ☐☐

Hvis du er usikker kan vi kontakte arbeidsgiver og få antall oppgitt, skriv i så fall ned arbeidsgiver og kontaktperson

Event. arbeidsgiver og kontaktperson: ____________________________

11) Mottar du svangerskapspenger?

☐ Ja ☐ Nei (gå videre til spørsmål 14)

12) Dersom du mottar svangerskapspenger, ber vi deg spesifisere nedenfor:

a) Jeg har mottatt svangerskapspenger fra (ddmmåååå): ☐☐ . ☐☐ . ☐☐☐☐

b) Jeg mottar svangerskapspenger i ☐☐☐ %

13) Årsak til at du mottar svangerskapspenger:

☐ Arbeid med kjemiske stoffer
☐ Fysisk slitsomt arbeid
☐ Arbeid med stressbelastning
☐ Psykososiale forhold

☐ Annet:________________________________

Eventuelle kommentarer:

____

____

____

FYSISK AKTIVITET OG TRENING

Statens råd for ernæring og fysisk aktivitet skiller mellom fysisk aktivitet og trening. Vennligst forhold deg til definisjonene nedenfor når du besvarer de neste spørsmålene.

***Fysisk aktivitet* definerer som "all kroppslig bevegelse produsert av skjelettmuskulatur som resulterer i en vesentlig økning i energiforbruket utover hvilenivå".**

14) Sosial- og helsedirektoratet anbefaler voksne å være i fysisk aktivitet 30 minutter daglig for å oppnå helsegevinst. Oppfyller du dette daglig?

☐ Ja ☐ Nei

***Trening* defineres som "fysisk aktivitet i fritiden som gjentas regelmessig over tid med målsetting å forbedre for eksempel form, prestasjon eller helse".**

15) Trener du regelmessig nå?

☐ Ja ☐ Nei *(gå videre til spørsmål 20)*

16) Hvis ja, hvor mange dager i uken trener du?

☐ 1 dag
☐ 2 dager
☐ 3 dager
☐ 4 dager
☐ 5 eller flere dager

17) Hvor lenge trener du vanligvis per økt?

☐ 0-30 min ☐ 30-60 min ☐ 60-90 min ☐ >90 min

18) På hvilken intensitet trener du vanligvis?

☐ Uten å bli svett eller andpusten (oppleves litt anstrengende)
☐ Blir svett og litt andpusten (oppleves anstrengende)
☐ Blir veldig svett og puster tungt (oppleves svært anstrengende)

19) Hvilken form for trening bedriver du nå? *(sett ett eller flere kryss)*

☐ Spesiell gymnastikk/ aerobic for gravide
☐ Aerobic/gymnastikk/ dans uten hopp og løp
☐ Aerobic/gymnastikk/ dans med hopp og løp
☐ Folkedans/rock/swing
☐ Sykling
☐ Rask gange/turgange
☐ Løping/jogging/ orientering/skigåing
☐ Ballspill/nettballspill
☐ Svømming
☐ Helsestudio/styrketrening
☐ Yoga/Pilates
☐ Kampsport
☐ Annet:_______________

20) Hvor ofte gjør du hjemmeøvelser for disse muskelgrupper?

	Aldri	1x/uke	2x/uke	3x/uke	>3x/uke
Magemuskler	☐	☐	☐	☐	☐
Ryggmuskler	☐	☐	☐	☐	☐
Bekkenbunnsmuskler (innvendige muskler rundt skjede, urinrør og endetarm)	☐	☐	☐	☐	☐

21) Hvor mange minutter bruker du hver dag på å sykle/gå/jogge til og fra arbeid?
(legg sammen tiden til og fra arbeidet)

☐ Ingen ☐ 20-30 min ☐ 30-60 min ☐ >60 min

SMERTER I RYGG OG BEKKEN

22) Har du hatt vondt i nedre del av ryggen (korsryggen) og/eller bekkenet i forbindelse med svangerskapet?

☐ Ja, smertene startet:
- ☐ Før dette svangerskapet
- ☐ Uke 0-20
- ☐ Uke 20-24
- ☐ Uke 24-28
- ☐ Uke 28-32
- ☐ Uke 32-36

☐ Nei (gå videre til spørsmål 29)

23) Opplevelse av korsrygg- og/eller bekkensmerter

- ☐ Ingen smerte
- ☐ Svak smerte
- ☐ Moderat smerte
- ☐ Sterk smerte
- ☐ Intens smerte
- ☐ Voldsom smerte

24) Har korsrygg- og /eller bekkensmertene endret seg fra forrige test?

- ☐ Bedre
- ☐ Uforandret
- ☐ Verre

25) Dersom du har bekkensmerter, hvor har du vondt?

- ☐ Foran i bekkenet (symfysen)
- ☐ Bak i bekkenet, på den ene siden
- ☐ Bak i bekkenet, på begge sider
- ☐ Både foran og bak i bekkenet på begge sider
- ☐ Både foran og bak i bekkenet på den ene siden

26) Våkner du av disse smertene om natta?

- ☐ Ja, ofte
- ☐ Ja, en sjelden gang
- ☐ Nei, aldri

27) Hvor mye påvirker korsrygg og/eller bekkensmertene dine daglige aktiviteter?
Vennligst sett kryss under et nummer mellom 0 (=ikke i det hele tatt) og 10 (=svært mye)

0	1	2	3	4	5	6	7	8	9	10
☐	☐	☐	☐	☐	☐	☐	☐	☐	☐	☐

28) Hvor sterke er dine smerter?
Vurder ditt nåværende smertenivå ved å sette ett kryss på hver av linjene under.

Hvor sterk er din smerte på det verste om morgenen, etter at du har stått opp?

Ingen smerte |———————————| **Uutholdelig smerte**

Ikke skriv her

Hvor sterk er din smerte på det verste om kvelden, før du legger deg?

Ingen smerte |———————————| **Uutholdelig smerte**

Ikke skriv her

7911

ID-nummer: ☐☐☐

29) Hvordan klarer du følgende aktiviteter nå i dag?
Angi etter hvert spørsmål graden av problemer du har med å gjennomføre aktiviteten, på grunn av rygg- og/eller bekkensmerter. Sett ett kryss på linjen etter hvert spørsmål.

Eksempel:

Uproblematisk ↓ |———————————| ↓ Umulig

Litt vanskelig - Vanskelig - Svært vanskelig

	**Uproblematisk** — **Umulig**	Ikke skriv her
Av- og påkledning uten hjelp……..	|————————|	☐☐☐
Gange / spasere, gå tur……………	|————————|	☐☐☐
Gå i trapper………………………	|————————|	☐☐☐
Sitte en lang stund…………………	|————————|	☐☐☐
Stå foroverbøyd………………….. (ex: over vaskeservant, stellebord)	|————————|	☐☐☐
Bære en bag (ca 6 kg)……………	|————————|	☐☐☐
Re seng……………………………	|————————|	☐☐☐
Løpe………………………………	|————————|	☐☐☐
Lett arbeid…………….........……	|————————|	☐☐☐
Tungt arbeid.............................	|————————|	☐☐☐
Tunge løft…………………………	|————————|	☐☐☐
Mosjon/sport/trening/trim………..	|————————|	☐☐☐

30) Har du fått behandling av fysioterapeut eller andre yrkesgrupper i dette svangerskapet for dine korsrygg- /bekkenplager? ☐ Ja ☐ Nei

7911

UFRIVILLIG LEKKASJE AV URIN

31) Har du ufrivillig urinlekkasje nå ved:

a) Hosting, nysing, latter	☐ Ja	☐ Nei
b) Fysisk aktivitet (løp og hopp)	☐ Ja	☐ Nei
c) Brå bevegelser, løft	☐ Ja	☐ Nei
d) Plutselig trang (problemer med å nå frem til toalettet i tide)	☐ Ja	☐ Nei

(Dersom du har svart nei på alle spørsmål 31, kan du gå til spørsmål 41)

32) Dersom du har symptomer både som i a, b eller c og som i d på spørsmål 31), hva er du mest plaget av?

☐ Lekkasje ved fysisk aktivitet mer enn lekkasje ved trang
☐ Lekkasje ved trang mer enn lekkasje ved fysisk aktivitet
☐ Like mye plaget av lekkasje ved trang som ved lekkasje ved fysisk aktivitet

33) Dersom du har urinlekkasje, hvor ofte lekker du? *(sett ett kryss)*

☐ Har ikke urinlekkasje
☐ Sjeldnere enn en gang per måned
☐ En eller flere ganger per måned
☐ En eller flere ganger per uke
☐ Hver dag og /eller natt

34) Hvor store mengder urin lekker du vanligvis om gangen? *(sett ett kryss)*

☐ Har ikke urinlekkasje *(gå videre til spørsmål 40)*
☐ Dråper
☐ Små skvetter
☐ Større mengder

35) Har lekkasjen av urin endret seg fra forrige test?

☐ Ja, jeg har blitt:
- ☐ Kontinent (ikke lekkasje)
- ☐ Nesten kontinent
- ☐ Bedre
- ☐ Uforandret
- ☐ Verre

☐ Nei

36) Bruker du truseinnlegg eller annen beskyttelse mot urinlekkasje?

☐ Alltid på dagtid
☐ Av og til på dagtid
☐ Alltid, også om natten
☐ Kun under fysisk aktivitet
☐ Aldri

37) Når startet din urinlekkasje?

- ☐ Før dette svangerskapet
- ☐ Uke 0-20
- ☐ Uke 20-24
- ☐ Uke 24-28
- ☐ Uke 28-32
- ☐ Uke 32-36

38) Opplevelse av urinlekkasje

- ☐ Uproblematisk
- ☐ Litt vanskelig
- ☐ Moderat vanskelig
- ☐ Vanskelig
- ☐ Svært vanskelig

39) Hvor mye påvirker urinlekkasjen dine daglige aktiviteter?
Vennligst sett kryss under et nummer mellom 0 (=ikke i det hele tatt) og 10 (=svært mye)

0	1	2	3	4	5	6	7	8	9	10
☐	☐	☐	☐	☐	☐	☐	☐	☐	☐	☐

40) Har du fått behandling av fysioterapeut, eller andre yrkesgrupper, for din urinlekkasje?

- ☐ Ja, jeg har fått:
  - ☐ Bekkenbunnstrening
  - ☐ Annet
- ☐ Nei

SYMPTOMER PÅ UNDERLIVSPROLAPS

41) Har du symptomer på underlivsprolaps dvs nedsynkning av underlivets/bekkenorganer. Det kan dreie seg om nedfall av: livmor, urinblære eller endetarm?

- ☐ Ja
- ☐ Nei, *(gå til spørsmål 45)*

42) Hvor lenge har du hatt symptomer på underlivsprolaps (utbulning i skjedeåpning og/eller tyngdefornemmelse)?

- ☐ Mindre enn ½ år
- ☐ ½ - 1 år
- ☐ 1-2 år
- ☐ Mer enn 2 år

43) Hemmer underlivsprolapset deg i det daglige liv?

- ☐ Nei, eller mindre enn 1 gang i måneden *(gå til spørsmål 45)*
- ☐ Ja, 1-3 ganger i måneden
- ☐ Ja, en til flere ganger i uken
- ☐ Ja, en til flere ganger daglig

44) I hvilke situasjoner hemmer underlivsprolapset deg?

- ☐ Ved arbeid i hjemmet
- ☐ Ved arbeid på arbeidsplassen
- ☐ Under innkjøp
- ☐ Ved fysiske utfoldelser (trening, dans)
- ☐ Under selskapeligheter
- ☐ Begrenser mine muligheter til å reise

45) Kan du føle en utbuling i skjedeåpningen?

- ☐ Nei, eller mindre enn 1 gang i måneden *(gå til spørsmål 47)*
- ☐ Ja, 1-3 ganger i måneden
- ☐ Ja, en til flere ganger i uken
- ☐ Ja, en til flere ganger daglig

46) Hvis du kan føle en utbuling i skjedeåpningen, er det et problem for deg?

- ☐ Ikke noe problem
- ☐ Lite problem
- ☐ Moderat problem
- ☐ Stort problem

47) Har du trykk eller tyngdefølelse i skjeden?

- ☐ Nei, eller mindre enn 1 gang i måneden *(gå til spørsmål 49)*
- ☐ Ja, 1-3 ganger i måneden
- ☐ Ja, en til flere ganger i uken
- ☐ Ja, en til flere ganger daglig

48) Hvis du har trykk eller tyngdefølelse i skjeden, er det et problem for deg?

- ☐ Ikke noe problem
- ☐ Lite problem
- ☐ Moderat problem
- ☐ Stort problem

UFRIVILLIG LEKKASJE AV AVFØRING

49) Har du problemer med å holde på…

	Ingen episoder siste 4 uker	1 episode siste 4 uker	Mer enn 1 episode siste 4 uker	1 eller flere episoder i uken	1 eller flere episoder om dagen
Fast avføring	☐	☐	☐	☐	☐
Væske- /løs avføring	☐	☐	☐	☐	☐
Luft	☐	☐	☐	☐	☐

50) Må du tilpasse hverdagen din på grunn av luft-/avføringslekkasjer?

☐ Aldri ☐ Sjelden ☐ Noen ganger ☐ Ukentlig ☐ Daglig

51) Bruker du bleie/bind eller plugg på grunn av avføringslekkasjer?

☐ Ja ☐ Nei

52) Bruker du medisiner mot ufrivillig lekkasje fra tarmen?

☐ Ja ☐ Nei

53) Kan du utsette avføring i 15 minutter etter første følelse av trang?

☐ Ja ☐ Nei

54) Har lekkasjen av luft-/avføring endret seg fra forrige test?

☐ Har ikke luft-/avføringslekkasje (gå videre til spørsmål 59)

☐ Ja, jeg har blitt:
- ☐ Kontinent (ikke lekkasje)
- ☐ Nesten kontinent
- ☐ Bedre
- ☐ Uforandret
- ☐ Verre

55) Når startet din luft-/avføringslekkasje?
Luft-/avføringslekkasjen startet:

☐ Før <u>dette</u> svangerskapet
☐ Uke 0-20
☐ Uke 20-24
☐ Uke 24-28
☐ Uke 28-32
☐ Uke 32-36

56) Opplevelse av

Avføringslekkasje:

- ☐ Uproblematisk
- ☐ Litt vanskelig
- ☐ Moderat vanskelig
- ☐ Vanskelig
- ☐ Svært vanskelig

Lekkasje av luft:

- ☐ Uproblematisk
- ☐ Litt vanskelig
- ☐ Moderat vanskelig
- ☐ Vanskelig
- ☐ Svært vanskelig

57) Hvor mye påvirker luft-/avføringslekkasjen dine daglige aktiviteter?
Vennligst sett kryss under et nummer mellom 0 (=ikke i det hele tatt) og 10 (=svært mye)

0	1	2	3	4	5	6	7	8	9	10
☐	☐	☐	☐	☐	☐	☐	☐	☐	☐	☐

58) Har du fått behandling av fysioterapeut, eller andre yrkesgrupper, for din luft-/avføringslekkasje?

- ☐ Ja, jeg har fått:
  - ☐ Bekkenbunnstrening
  - ☐ Annet
- ☐ Nei

ANDRE PLAGER I NÅVÆRENDE SVANGERSKAP

59) Dersom du har hatt svangerskapskvalme i dette svangerskapet, ber vi deg oppgi dato for når svangerskapskvalmen startet og hvor lenge den varte:

Svangerskapskvalmen startet (dd.mm.åååå) ☐☐.☐☐.☐☐☐☐

Svangerskapskvalmen varte til (dd.mm.åååå) ☐☐.☐☐.☐☐☐☐

60) Har du fått strekkmerker i dette svangerskapet?

- ☐ Ja
- ☐ Nei *(gå til spørsmål 62)*

61) Dersom du har fått strekkmerker i dette svangerskapet, hvor er disse?

- ☐ Magen
- ☐ Lår
- ☐ Bryster
- ☐ Overarmer

SPØRSMÅL OM VELVÆRE

62) Hvordan har du vanligvis FØLT DEG den siste uken?

- ☐ I usedvanlig godt humør
- ☐ I svært godt humør
- ☐ For det meste i godt humør
- ☐ Humøret har variert
- ☐ For det meste i dårlig humør
- ☐ I svært dårlig humør

63) Hvor ofte har du vært PLAGET av SYKDOM, FYSISKE PLAGER, SMERTE eller VERK den siste uken?

- ☐ Hver dag
- ☐ Nesten hver dag
- ☐ Omtrent halvparten av tiden
- ☐ Av og til, men mindre enn halvparten av tiden
- ☐ Sjelden
- ☐ Ikke i det hele tatt

64) Har du følt deg NEDSTEMT eller NEDFOR den siste uken?

- ☐ Ja, i den grad at jeg har følt det som livet ikke er til å leve
- ☐ Ja, så jeg nesten ikke har brydd meg om noen ting
- ☐ Ja, meget nedfor nesten hver dag
- ☐ Ja, ganske nedfor ved flere anledninger
- ☐ Ja, litt nedfor av og til
- ☐ Nei, ikke nedfor i det hele tatt

65) Har du hatt GOD KONTROLL over dine HANDLINGER, dine TANKER og FØLELSER den siste uken?

- ☐ Ja, absolutt
- ☐ Ja, for det meste
- ☐ Vanligvis
- ☐ Ikke helt
- ☐ Nei, og det er forstyrrende
- ☐ Nei, og det er svært forstyrrende

66) Har du vært plaget av NERVØSITET eller av dine "NERVER" den siste uken?

- ☐ Ekstremt mye – i den grad at jeg ikke har kunnet arbeide eller ta hånd om andre gjøremål
- ☐ Svært mye
- ☐ En god del
- ☐ Noe – nok til å plage meg
- ☐ Litt
- ☐ Ikke i det hele tatt

67) Hvor ENERGISK, SPREK eller VITAL har du vært den siste uken?

- ☐ Virkelig full av energi, svært sprek
- ☐ Ganske energisk det meste av tiden
- ☐ Min energi og vitalitet har variert en god del
- ☐ Vanligvis lite energisk
- ☐ Svært lite energi det meste av tiden
- ☐ Ingen energi og vitalitet i det hele tatt – jeg har kjent meg utladet og tom

68) Jeg har følt meg TRIST og vært LEI MEG den siste uken

- ☐ Ikke i det hele tatt
- ☐ Ved svært få anledninger
- ☐ Av og til
- ☐ Ganske ofte
- ☐ For det meste
- ☐ Hele tiden

69) Har du følt deg ANSPENT den siste uken?

- ☐ Ekstremt anspent nesten hele tiden eller hele tiden
- ☐ Svært anspent mesteparten av tiden
- ☐ Ikke vanligvis, men ganske anspent ved flere anledninger
- ☐ Litt anspent av og til
- ☐ Ikke spesielt anspent
- ☐ Ikke anspent i det hele tatt

70) Hvor LYKKELIG, FORNØYD eller TILFREDS med livet har du vært den siste uken?

- ☐ Usedvanlig lykkelig – kunne ikke være mer fornøyd og tilfreds
- ☐ For det meste svært lykkelig
- ☐ Vanligvis fornøyd og tilfreds
- ☐ Noen ganger ganske fornøyd andre ganger ganske misfornøyd
- ☐ Generelt utilfreds og misfornøyd
- ☐ Alltid eller for det meste svært utilfreds og ulykkelig

71) Har du følt deg så FRISK at du har kunnet GJØRE DET DU LIKER Å GJØRE, eller det du MÅTTE GJØRE, den siste uken?

- ☐ Ja, så absolutt
- ☐ For det meste
- ☐ Helseproblemer har satt begrensninger for meg på noen viktige områder
- ☐ Jeg har bare vært frisk nok til å ta vare på meg selv
- ☐ Jeg har trengt noe hjelp for å klare meg
- ☐ Jeg har trengt hjelp til nesten alt jeg skulle gjøre

72) Har du følt deg så TRIST, MOTLØS eller UTEN HÅP at du har lurt på om livet var verd å leve den siste uken?

- ☐ Ekstremt mye, i den grad at jeg var i ferd med å gi opp
- ☐ Svært mye
- ☐ En god del
- ☐ Noe – nok til å plage meg
- ☐ Litt
- ☐ Ikke i det hele tatt

73) Jeg har følt meg FRISK og UTHVILT når jeg har våknet den siste uken

- [ ] Ikke i det hele tatt
- [ ] Ved svært få anledninger
- [ ] Av og til
- [ ] Ganske ofte
- [ ] For det meste
- [ ] Hele tiden

74) Har du vært BEKYMRET eller ENGSTELIG FOR DIN HELSE den siste uken?

- [ ] Ekstremt mye
- [ ] Svært mye
- [ ] Noe, men ikke mye
- [ ] Praktisk talt aldri
- [ ] Ikke i det hele tatt

75) Har du hatt grunn til å tro at du var i ferd med å MISTE FORSTANDEN eller MISTE KONTROLLEN OVER DINE FØLELSER, TANKER og HANDLINGER den siste uken?

- [ ] Ikke i det hele tatt
- [ ] Bare litt
- [ ] Litt, men ikke så mye at det har bekymret eller uroet meg
- [ ] En del og det har bekymret meg litt
- [ ] En god del og det har uroet meg ganske mye
- [ ] Ja, svært ofte og jeg er svært bekymret

76) Dagliglivet har vært FYLT MED TING SOM INTERESSERER meg den siste uken

- [ ] Ikke i det hele tatt
- [ ] Ved svært få anledninger
- [ ] Av og til
- [ ] Ganske ofte
- [ ] For det meste
- [ ] Hele tiden

77) Har du følt deg AKTIV og ENERGISK eller SLAPP og GIDDESLØS den siste uken?

- [ ] Svært aktiv og energisk hver dag
- [ ] For det meste aktiv og energisk – aldri ordentlig slapp eller giddesløs
- [ ] Forholdsvis aktiv og energisk - sjelden slapp og giddesløs
- [ ] Forholdsvis slapp og giddesløs - sjelden aktiv og energisk
- [ ] For det meste slapp og giddesløs – aldri riktig aktiv og energisk
- [ ] Svært slapp og giddesløs hver dag

78) Har du vært ENGSTELIG, BEKYMRET eller UTE AV BALANSE den siste uken?

- [ ] Så avgjort – i den grad at jeg har kjent meg dårlig eller nesten syk
- [ ] Svært mye
- [ ] En god del
- [ ] Noe – nok til å bekymre meg
- [ ] Litt
- [ ] Ikke i det hele tatt

79) Jeg har vært FØLELSESMESSIG STABIL og SIKKER PÅ MEG SELV den siste uken

- ☐ Ikke i det hele tatt
- ☐ Ved svært få anledninger
- ☐ Av og til
- ☐ Ganske ofte
- ☐ For det meste
- ☐ Hele tiden

80) Har du følt deg AVSLAPPET og ROLIG eller STRESSET og ANSPENT den siste uken?

- ☐ Hele tiden avslappet og rolig
- ☐ For det meste avslappet og rolig
- ☐ Vanligvis rolig, men av og til ganske anspent
- ☐ Vanligvis anspent, men ved enkelte tilfeller ganske avslappet
- ☐ For det meste stresset og anspent
- ☐ Hele tiden stresset og anspent

81) Jeg har følt meg MUNTER og I GODT HUMØR den siste uken

- ☐ Ikke i det hele tatt
- ☐ Ved svært få anledninger
- ☐ Av og til
- ☐ Ganske ofte
- ☐ For det meste
- ☐ Hele tiden

82) Jeg har følt meg TRØTT, SLITEN eller UTSLITT den siste uken

- ☐ Ikke i det hele tatt
- ☐ Ved svært få anledninger
- ☐ Av og til
- ☐ Ganske ofte
- ☐ For det meste
- ☐ Hele tiden

83) Har du vært under eller følt deg utsatt for PÅKJENNING, STRESS eller PRESS den siste uken?

- ☐ Ja, nesten mer enn jeg har kunnet klare eller holde ut
- ☐ Ja, under en god del press
- ☐ Ja, noe – mer enn vanlig
- ☐ Ja, noe – omtrent som vanlig
- ☐ Ja, litt
- ☐ Nei, ikke i det hele tatt

ULIKE UTSAGN OG PÅSTANDER

84) Hvor viktig er det for en gravid kvinne å…
Sett et kryss for hver linje

	Ikke viktig i det hele tatt				Veldig viktig
a)...få en god natts søvn......................	☐	☐	☐	☐	☐
b)...ikke røyke......................................	☐	☐	☐	☐	☐
c)…ikke drikke mye alkohol.................	☐	☐	☐	☐	☐
d)…hvile og slappe av..........................	☐	☐	☐	☐	☐
e)…unngå å bekymre seg mye............	☐	☐	☐	☐	☐
f)…utføre daglige gjøremål..................	☐	☐	☐	☐	☐
g)…unngå å bli overvektig...................	☐	☐	☐	☐	☐
h)…trene regelmessig..........................	☐	☐	☐	☐	☐
i)…unngå fettholdig mat.......................	☐	☐	☐	☐	☐
j)…ha en aktiv livsstil...........................	☐	☐	☐	☐	☐

85) Kryss av i hvilken grad du er enig eller uenig i de følgende påstandene:

a) En gravid kvinne bør trene mindre enn en kvinne som ikke er gravid.

☐ Helt enig ☐ Nokså enig ☐ Nokså uenig ☐ Helt uenig ☐ Vet ikke

b) Når man er gravid, kan man med god samvittighet la være å trene. Det er viktigere med ro og hvile.

☐ Helt enig ☐ Nokså enig ☐ Nokså uenig ☐ Helt uenig ☐ Vet ikke

c) Det er viktig å trene regelmessig under svangerskapet.

☐ Helt enig ☐ Nokså enig ☐ Nokså uenig ☐ Helt uenig ☐ Vet ikke

d) Når man er gravid, bør man ikke anstrenge seg så hardt under treningen at man blir helt utmattet.

☐ Helt enig ☐ Nokså enig ☐ Nokså uenig ☐ Helt uenig ☐ Vet ikke

e) Hvis man trener, bør man redusere treningsmengden etter hvert i svangerskapet

☐ Helt enig ☐ Nokså enig ☐ Nokså uenig ☐ Helt uenig ☐ Vet ikke

f) Hvis man ikke har trent før svangerskapet, trenger man ikke begynne å trene i svangerskapet.

☐ Helt enig ☐ Nokså enig ☐ Nokså uenig ☐ Helt uenig ☐ Vet ikke

86) Kryss av i hvilken grad du er enig eller uenig i disse utsagnene:
"Trening under svangerskapet…

a) …gir mer energi og krefter til å takle de omstillinger som svangerskapet medfører".

☐ Helt enig ☐ Nokså enig ☐ Nokså uenig ☐ Helt uenig ☐ Vet ikke

b) …bedrer det generelle humøret".

☐ Helt enig ☐ Nokså enig ☐ Nokså uenig ☐ Helt uenig ☐ Vet ikke

c) …har en stressreduserende effekt".

☐ Helt enig ☐ Nokså enig ☐ Nokså uenig ☐ Helt uenig ☐ Vet ikke

d) …hjelper meg å kontrollere vekten".

☐ Helt enig ☐ Nokså enig ☐ Nokså uenig ☐ Helt uenig ☐ Vet ikke

87) Hvor sannsynlig tror du det er at regelmessig trening under svangerskapet kan:

a) …redusere sjansene for svangerskapsforgiftning?

☐ Svært usannsynlig ☐ Ganske usannsynlig ☐ Ganske sannsynlig ☐ Svært sannsynlig ☐ Vet ikke

b) …være med på å forebygge unødig vektøkning?

☐ Svært usannsynlig ☐ Ganske usannsynlig ☐ Ganske sannsynlig ☐ Svært sannsynlig ☐ Vet ikke

c) …redusere sjansene for å utvikle svangerskapsdiabetes (sukkersyke)?

☐ Svært usannsynlig ☐ Ganske usannsynlig ☐ Ganske sannsynlig ☐ Svært sannsynlig ☐ Vet ikke

d) …redusere sjansene for svangerskapsdepresjoner?

☐ Svært usannsynlig ☐ Ganske usannsynlig ☐ Ganske sannsynlig ☐ Svært sannsynlig ☐ Vet ikke

e) …forebygge svangerskapsplager som leggkramper eller hevelser i beina?

☐ Svært usannsynlig ☐ Ganske usannsynlig ☐ Ganske sannsynlig ☐ Svært sannsynlig ☐ Vet ikke

f) …forebygge svangerskapsplager som halsbrann eller kvalme?

☐ Svært usannsynlig ☐ Ganske usannsynlig ☐ Ganske sannsynlig ☐ Svært sannsynlig ☐ Vet ikke

g) …forebygge rygg- og bekkensmerter?

☐ Svært usannsynlig ☐ Ganske usannsynlig ☐ Ganske sannsynlig ☐ Svært sannsynlig ☐ Vet ikke

h) …øke sannsynligheten for å få en kortere og lettere fødsel?

☐ Svært usannsynlig ☐ Ganske usannsynlig ☐ Ganske sannsynlig ☐ Svært sannsynlig ☐ Vet ikke

i) …forkorte tiden det tar å komme seg etter fødselen?

☐ Svært usannsynlig ☐ Ganske usannsynlig ☐ Ganske sannsynlig ☐ Svært sannsynlig ☐ Vet ikke

EPWORTH SLEEPINESS SCALE

88) Se for deg de nedenfor nevnte situasjoner. Hvor sannsynlig er det at du kan "duppe av" eller sovne i slike situasjoner?
(Selv om du ikke har vært i slike situasjoner nylig, prøv å forestille deg hva som mest sannsynlig ville ha skjedd)

Bruk følgende skala til å beskrive hva som er mest riktig for deg:
0= Ville aldri sovne/ duppe av.
1= Liten mulighet for å sovne/ duppe av.
2= Moderat mulighet for å sovne/ duppe av.
3= Stor mulighet for å sovne/ duppe av.

**Situasjoner:**	**Sannsynlighet for å sovne/duppe av:**			
	0	1	2	3
a) Sitter og leser	☐	☐	☐	☐
b) Sitter inaktiv på et offentlig sted (for eksempel på teater eller i et møte)	☐	☐	☐	☐
c) Er passasjer i bil en time uten stopp	☐	☐	☐	☐
d) Legger deg ned i løpet av dagen når omstendighetene tillater det	☐	☐	☐	☐
e) Sitter og snakker med noen	☐	☐	☐	☐
f) Sitter stille etter lunsj, uten alkohol	☐	☐	☐	☐
g) Kjører bil, men stopper i fem minutter på grunn av kø	☐	☐	☐	☐

EVALUERING FRA TRENINGSGRUPPEN

Besvares KUN av deltagerne i treningsgruppen

89) Har du klart å gjennomføre trening 3 ganger per uke hele denne perioden? *(To ganger i gruppe + en gang hjemme, eventuelt to ganger hjemme)*

- ☐ Ja *(gå til spørsmål 91)*
- ☐ Nei

90) Dersom du ikke har klart å gjennomføre dette, ønsker vi at du spesifiserer nærmere nedenfor hvorfor treningen (gruppe- og egentrening) var vanskelig å gjennomføre: *(Sett ett eller flere kryss)*

- ☐ Har ikke tid
- ☐ Har ikke motivasjon / interesse
- ☐ Smerter
- ☐ Ugunstig tidspunkt for gruppetreningen
- ☐ Vanskelig å komme seg til / fra treningslokalet
- ☐ Annet:________________________________

91) Hvor fornøyd er du med treningstilbudet du fikk ved å delta i prosjektet?

- ☐ Misfornøyd
- ☐ Litt misfornøyd
- ☐ Moderat fornøyd
- ☐ Fornøyd
- ☐ Svært fornøyd

92) Evaluering av gruppetreningen:

Var intensiteten?	☐ Høy	☐ Passe	☐ Lav
Valg av øvelser i utholdenhetsdelen?	☐ Vanskelig	☐ Passe	☐ Lett
Har timen tilfredsstilt forventningene?	☐ Ja	☐ Nei	

93) Evaluering av egentreningen:

Var øvelsene?	☐ Vanskelig	☐ Passe	☐ Lett
Var hele programmet?	☐ For tidkrevende	☐ Passe	

94) Tror du treningen har vært bra for deg og svangerskapet?

☐ Ja ☐ Nei ☐ Vet ikke

95) Synes du denne treningen er et tilbudet som bør gis alle gravide i framtiden?

☐ Ja ☐ Nei ☐ Vet ikke

96) Har du noen kommentarer du ønsker å formidle til oss (positive eller negative erfaringer med treningen), så skriv det gjerne ned: *(Bruk gjerne baksiden av arket)*

____

____
