# Supplementary material for: Exercise Training and Weight Gain in Obese Pregnant Women: A Randomized Controlled Trial (ETIP Trial)
Source: PLoS Med. 2016 Jul 26;13(7):e1002079. doi: 10.1371/journal.pmed.1002079 (PMC4961392; doi:10.1371/journal.pmed.1002079)
Supplement: S11 Text — (DOCX) [file pmed.1002079.s016.docx]

To whom it may concern

Deres ref.: Vår ref.:ER/BW Dato: 02.03.2016

**Regular exercise during pregnancy. Good for overweight women (7-370-00/08A)**

We hereby confirm that the project “Regular exercise during pregnancy. Good for overweight women” (7-370-00/08A) has been financed by The Norwegian Fund for Post-Graduate Training in Physiotherapy with NOK 2 800 000 for the period of 23.10.2010-23.10.2016.

Best regards


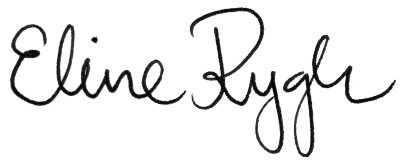

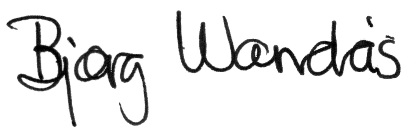

Eline Rygh

Head of office Bjørg Wandås

senior secretary
