# Supplementary material for: Exercise Training and Weight Gain in Obese Pregnant Women: A Randomized Controlled Trial (ETIP Trial)
Source: PLoS Med. 2016 Jul 26;13(7):e1002079. doi: 10.1371/journal.pmed.1002079 (PMC4961392; doi:10.1371/journal.pmed.1002079)
Supplement: S12 Text — (PDF) [file pmed.1002079.s017.pdf]

NTNU Samarbeidsorganet Helse Midt-Norge og NTNU HELSE MIDT-NORGE

To whom it may concern

Senior Executive Officer
Mai Hege Stokke
Tel: +47 93 63 33 80
E-mail: mai.h.stokke@ntnu.no

Our date:
March 2, 2016

Declaration of funding from The Liaison Committee between the Central Norway Regional Health Authority (RHA) and the Norwegian University of Science and Technology (NTNU)

This is to confirm that The Liaison Committee between the Central Norway Regional Health Authority (RHA) and the Norwegian University of Science and Technology (NTNU), is funding a Ph.D. grant for Kirsti Krohn Garnæs, for the research project "Reducing pregnancy risks with exercise in obese women". The Ph.D. grant covers 75 % work load for a 4-year period, and was awarded from 2013.

Yours sincerely,

Mai Hege Stokke

Mai Hege Stokke
Senior Executive Officer

Liaison Committee between the Central Norway Regional Health Authority (RHA)
and the Norwegian University of Science and Technology (NTNU)

Postadresse
Medisinsk teknisk forskningssenter
7491 Trondheim
samarbeidsorganet@medisin.ntnu.no

Besøksadresse
Olav Kyrres gt 9
7491 Trondheim
www.medisin.ntnu.no

Org. nr. 974 767 880
