# Supplementary material for: Exercise Training and Weight Gain in Obese Pregnant Women: A Randomized Controlled Trial (ETIP Trial)
Source: PLoS Med. 2016 Jul 26;13(7):e1002079. doi: 10.1371/journal.pmed.1002079 (PMC4961392; doi:10.1371/journal.pmed.1002079)
Supplement: S13 Text — (DOC) [file pmed.1002079.s018.doc]

**Forespørsel om å gi biologisk materiale til forskningsbiobank**

**Formål**

Forskningsbiobanken tar vare på biologisk materiale (for eksempel blod, vev og urin) som kan benyttes til medisinsk forskning. Formålet er å gi økt kunnskap om betydningen av trening i graviditeten, for derved å bidra til bedre forebygging, diagnostikk og behandling. I forbindelse med at du er med i studien ”Trening i svangerskapet for overvektige”, forespørres du om å gi materiale som stammer fra deg og ditt foster/barn. Vi ønsker å ta blodprøver av deg i starten av svangerskapet (rundt uke 14), på slutten av svangerskapet (rundt uke 34), og 3 måneder etter fødsel, samt fra navlestrengen idet barnet blir født. Noe av blodet som taes blir analysert med en gang, mens noe blir frosset ned for senere analysering. Det er overskudds-materialet fra disse prøvene som skal inngå i biobanken.

**Biologisk materiale og helseopplysninger**

All bruk av biologisk materiale og helseopplysninger i forskning vil skje i form av prosjekter, som først må godkjennes av Regional komité for medisinsk og helsefaglig forskningsetikk (REK). I enkelte tilfeller vil REK kunne komme fram til at det er behov for å innhente nytt samtykke for å benytte det innsamlede materialet. Materialet vil bli oppbevart og brukt så lenge det er noe igjen, og oppbevaring og bruk blir utført i samsvar med Helseforskningsloven og annet relevant lovverk.

**Bruk av journalopplysninger**

Vi ber om din tillatelse til å bruke opplysninger fra din pasientjournal når dette er nødvendig. Det kan være nærmere beskrivelse av den aktuelle tilstanden, hvordan den har utviklet seg, opplysninger om tidligere sykdommer, forekomst av tilsvarende tilstander i nær familie, hvilken behandling som er gitt og hvordan den har virket.

**Kobling av registre**

I enkelte prosjekt kan det være nødvendig å sammenstille resultatene med data fra store befolkningsundersøkelser (for eksempel Helseundersøkelsen i Nord-Trøndelag). Noen ganger kan det også være nødvendig å innhente tilleggsinformasjon som finnes i følgende helseregistre:

l. Dødsårsaksregisteret

2. Kreftregisteret

3. Medisinsk fødselsregister

4. Meldingssystem for smittsomme sykdommer

5. Det sentrale tuberkuloseregisteret

6. System for vaksinasjonskontroll

7. Forsvarets helseregister

8. Andre registre (ved bruk av andre registre skal REK og Datatilsynet vurdere behovet for innhenting av nytt samtykke).

Hvilke opplysninger som kan hentes fra helseundersøkelser eller helseregistre, vil være avgrenset av hvilke opplysninger som finnes lagret i disse. Vi ber om din tillatelse til å innhente tilleggsinformasjon fra de nevnte registre.

**Databehandling/taushetsplikt og formell godkjenning**

Alle som behandler biologisk materiale og opplysninger om dette, er underlagt taushetsplikt i henhold til Forvaltningsloven § 13 og Helsepersonelloven § 21. Det meste av forskningen vil foregå i Midt-Norge, men i enkelte tilfeller kan det være nødvendig å overføre deler av materialet til forskere andre steder i landet, eventuelt i utlandet. Det vil uansett ikke bli utlevert identifiserbare personopplysninger til forskere i utlandet. Opprettelsen av forskningsbiobanken er godkjent av Regional komité for medisinsk og helsefaglig forskningsetikk Midt-Norge.

**Dine rettigheter**

Det er frivillig om du vil tillate at biologisk materiale kan bli brukt til forskning eller ikke. Dersom du gir ditt skriftlige samtykke, kan du når som helst kreve å få vite hvilket materiale som finnes av deg, hva det er brukt til, samt hvilke resultater som har kommet ut av forskningen. Hvorvidt du velger å samtykke eller ikke, har ingen betydning for videre behandling ved sykehuset. Ønsker du å trekke tilbake et avgitt samtykke, kan du når som helst benytte følgende adresser/telefonnummer for å få fjernet og destruert materialet, uten å måtte begrunne dette nærmere:

Trine Moholdt

NTNU, Det medisinske fakultet

Institutt for sirkulasjon og bildediagnostikk

Postboks 8905
7491 Trondheim

Tlf: 97098594

E-postadr: trine.moholdt@ntnu.no

Dersom du ønsker å samtykke, ber vi deg signere vedlagte samtykkeerklæring.

Med vennlig hilsen,

Trine Moholdt,

Prosjektleder for studien ”Effektiv trening i svangerskapet for overvektige”

Jeg har lest informasjonsskrivet og har hatt anledning til å stille spørsmål. Jeg samtykker i å avgi materiale til forskningsbiobanken, at det kan brukes opplysninger fra min pasientjournal og at data kan kobles mot helseregistre.

Sted: . . . . . . . . . . . . . . . . . . . . . . . . . . . . . . . . . . . . . . . . . . .

Dato: . . . . . . . . . . . . . . . . . . . . . . . . . . . . . . . . . . . . . . . . . . .

Navn, blokkbokstaver: . . . . . . . . . . . . . . . . . . . . . . . . . . . . . . .

Underskrift: . . . . . . . . . . . . . . . . . . . . . . . . . . . . . . . . . . . . . . . . . . .
