# Supplementary material for: Exercise Training and Weight Gain in Obese Pregnant Women: A Randomized Controlled Trial (ETIP Trial)
Source: PLoS Med. 2016 Jul 26;13(7):e1002079. doi: 10.1371/journal.pmed.1002079 (PMC4961392; doi:10.1371/journal.pmed.1002079)
Supplement: S14 Text — (PDF) [file pmed.1002079.s019.pdf]

REK
DE REGIONALE KOMITEER FOR MEDISINSK OG HELSEFAGLIG FORSKNINGSETIKK

To Whom It May Concern

Reference:
2010/1522

Date:
31.01.2011

Exercise training in pregnancy.

Project leader: Trine Moholdt

The Regional Research Ethics Committee in Medicine, Central Norway, evaluated and accepted the prosject on June 18, 2010.

Sincerely

Sven Erik Gisvold /S/
Professor
Leader of the Committee

Hilde Eikemo
Hilde Eikemo
Advisor
Secretary of the Committee
